# Genome-wide analysis and identification of nuclear factor Y gene family in switchgrass (*Panicum virgatum L.*)

**DOI:** 10.1186/s12864-024-11092-6

**Published:** 2024-12-20

**Authors:** Hadia Hussain, Noor Fatima, Muhammad Sajid, Iqra Mehar, Maryam Noor, Kotb A. Attia, Yaser M. Hafez, Khaled Abdelaal, Tawaf Ali Shah

**Affiliations:** 1 Department of Biotechnology, University of Okara, Okara, Pakistan; 2 Key Laboratory of Molecular Epigenetics of MOE, Northeast Normal University, Changchun, 130024 China; 3 Department of Botany, Government College University Faisalabad, Faisalabad, Pakistan; 4 Department of Biochemistry, College of Science, King Saud University, P.O. Box 2455, Riyadh, 11451 Saudi Arabia; 5 EPCRS Excellence Center, Plant Pathology and Biotechnology Lab, Agric. Botany Dept., Fac. Agric, Kafrelsheikh Univ, Kafr el-Sheikh, 33516 Egypt; 6 College of Agriculture Engineering and Food Science, Shandong University of Technology, Zibo, 255000 China

**Keywords:** Switchgrass, *NF-Y* gene family, Expression analysis, Genome-wide analysis

## Abstract

NF-Y is a class of heterotrimeric transcription factor composed of three subunits; NF-YA, NF-YB, and NF-YC. This complex binds to the CCAAT box found in eukaryotic promoters and is involved in the plant development and proliferation at various stages. Although many studies were conducted on *NF-Y* gene family in various species, but no study has been reported yet in switchgrass (*Panicum virgatum L.*). In this study, 47 *PvNF-Y* genes (17 *PvNF-YA*, 18 *PvNF-YB*, and *12 PvNF-YC*) have been identified and named according to their subfamily. Chromosome location analysis revealed that all 47 *PvNF-Y* genes are randomly distributed across nine chromosomes. Moreover, multiple sequence alignment showed the DNA-binding domain and NF-YA/NFYB interacting domains flanking with non-conserved domains. In addition, prediction of functional similarities among *PvNF-Ys* genes phylogenetic tree was constructed corresponding to *Arabidopsis*. The gene structure, conserved domains and motifs analysis of *PvNF-Ys* genes demonstrated their specificity and functional conservation. *Cis*-regulatory elements analysis identified numerous key CREs that are significantly associated with light, hormone, stress and plant development responses. Expression profiling indicated higher expression levels of many *PvNF-YA* genes during drought and heat stress. Additionally, qRT-PCR analysis showed that some *PvNF-Ys* genes have high expression level in root. In conclusion, the findings of this study could provide a foundation for further cloning and functional analysis of *NF-Y* genes in switchgrass.

**Supplementary Information:**

The online version contains supplementary material available at 10.1186/s12864-024-11092-6.

## Introduction

Transcription factors (TFs) can bind to *cis*-acting sites in eukaryotic gene promoter regions, thereby regulating the activation or inhibition of transcription and gene expression at particular growth and development stages [1]. Nuclear Factor Y (NF-Y), also referred to as heme activator protein (HAP) or CCAAT-binding factor (CBF), is a heterotrimeric transcription factor found across eukaryotes. In plants, NF-Y consists of three conserved subunits: NF-YA (HAP2), NF-YB (HAP3/CBF-A), and NF-YC (HAP5/CBF-C). These subunits work together to regulate various biological processes by modulating gene expression at critical developmental stages and in response to environmental cues [2]. Initially, the NF-YB and NF-YC subunits form a dimer in the cytoplasm. Then, this dimer translocates to the nucleus, where it associates with the NF-YA subunits to create a heterotrimeric complex. Finally, NF-Y heterotrimer subsequently binds to the CCAAT-box in the promoter regions to modulate the transcription of specific genes [2, 3]. In plants, subfamilies of NF-Y are essential for a wide array of developmental processes, including flowering time regulation [4], bud and root differentiation [5], embryogenesis [6], seed germination [6, 7] and chloroplast biogenesis [8]. Beyond development, these subfamilies are also essential for conferring tolerance to various abiotic stresses, such as drought, temperature extremes and salinity [9–12].

Recent studies have investigated the *NF-Y* gene family in various species, such as *Arabidopsis thaliana* [13], *Oryza sativa* [14], *Brassica napus* [15], *Glycine max* [16], *Brassica rapa* [17], *Cucumis melo* [18], *Prunus persica* [19], *Solanum lycopersicum* [20], *Sorghum* [21], *foxtail millet* [22], *Vitis vinifera* [23], and *Hordeum vulgare* [24], indicating the significance of the *NF-Y* gene family in physiological ecology and abiotic stress responses across these diverse species. Previous studies have reported that *AtNF-YA1* is linked to post-germinative growth under salt stress in *Arabidopsis thaliana* [10]. Over-expression of *AtNF-YA5* leads to enhanced resistance to drought by triggering stress-responsive genes in *Arabidopsis thaliana* [8]. *AtNF-YB1* functions through a previously undescribed mechanism to confer improved drought performance in *Arabidopsis* [25]. *OsNF-YC5* negatively affects salt tolerance in *Oryza sativa* under abiotic stress [26]. Over-expression of *Ginkgo biloba* substantially enhances heat shock factor expression (GbHSF) in callus tissue under heat stress, indicating that *GbNF-YA6* plays a critical role in improving plant heat tolerance [27].

Switchgrass (*Panicum virgatum L.*) is a perennial warm-season C4 (NAD-malic enzyme) type grass that has a bunchgrass-like appearance and native to the tallgrass prairies of North America [28, 29]. Switchgrass being a C4 plant is a member of poaceae family and is considered a promising biofuel crop because of its high productivity rate, substantial genetic diversity, and broad native geographic distribution [30, 31]. Compared to traditional crops, switchgrass needs minimal management and utilizes resources more efficiently, particularly water, making it ideal for sustainable bioenergy production [32]. Switchgrass can confer resistance to salinity, drought, and inadequate nutrition [33]. Recent advancements in genetic linkage mapping, gene expression, genome high-throughput sequencing, and assembly have established switchgrass as a model species for energy crops [34, 35]. Additionally, microRNAs and long non-coding RNAs were sequenced and analyzed to understand how switchgrass regulates its response to drought stress [36, 37].

Although *NF-Y* gene family has been widely reported in various plant species. However, its roles and functions in switchgrass remain poorly understood till now. In this study, many bioinformatics tools were used to analyze the *NF-Y* gene family in switchgrass genome. To identify members of *NF-Y* genes, a deterministic approach was used including multiple sequence alignment, gene structure, conserved domains, motifs analysis, chromosome distribution, comparative phylogenetic analysis, *cis*-regulatory analysis, enrichment and expression pattern analysis. This study could provide valuable insights for identifying candidate *NF-Y* genes involved in regulating growth, development, and response to abiotic stresses in switchgrass.

## Materials and methods

### Identification of *NF-Y* gene family in the switchgrass

NF-Y protein sequences of *Arabidopsis thaliana* were downloaded from TAIR (https://www.arabidopsis.org/browse/genefamily/index.jsp/ accessed on 03 April 2024), and the genome file of switchgrass (*Panicum virgatum L.*) was downloaded from Phytozome v13.0 (https://phytozome-next.jgi.doe.gov/ was accessed on 03 April 2024). To identify NF-Y proteins in switchgrass, BLAST-P (https://blast.ncbi.nlm.nih.gov/Blast.cgi/ accessed on 05 April 2024) was performed with E-value ≤ 1e-5 as default parameter, and PvNF-Ys proteins were successfully matched to corresponding AtNF-Ys proteins (File S1). The physicochemical properties were calculated through ExPASy program (https://web.expasy.org/protparam/ accessed on 08 April 2024), to predict the length of protein, theoretical isoelectric point (pI), and molecular weight (MW) [38]. In silico subcellular localization was conducted by WOLF PSORT (https://wolfpsort.hgc.jp/ accessed on 09 April 2024) [39].

### Functional domain and conserved motif analysis

Additionally, all candidate PvNF-Ys proteins were further analyzed to confirm the presence of conserved domains using the NCBI-Batch CD Search (accessed on 07 April 2024). Finally, only the genes containing conserved patterns of domains were selected for subsequent analysis. Further, the conserved motifs of the identified *PvNF-Ys* genes were predicted by using MEME-suite tool (https://meme-suite.org/tools/meme, accessed on 12 April 2024) to understand the structure and peptide sequence of *PvNF-Ys* genes with default values and maximum 10 number of motifs set or other variables [40].

### Multiple sequence alignment and phylogenetic analysis

To further identify the evolutionary relationship between NF-Y proteins in switchgrass. Multiple sequence alignment was performed by using ClustalW program with default parameters. Then, phylogenetic tree was constructed using the neighbor-joining (NJ) method in MEGA 11 software. The tree was then annotated and beautified by using the iTOL tool (accessed on 10 April 2024) [41].

### Gene structure, and chromosomal distribution analysis

To predict the gene structure and chromosomal distribution of all the predicted PvNF-Ys proteins, we used TBtool for identification and visualization [42]. Each *PvNF-Ys* gene was mapped to switchgrass chromosomes according to the gene number and location, where duplicated gene pairs were determined on the bases of higher sequence identity (> 70–80) and stronger evolutionary relationships and linked with a colour line.

### *Cis*-regulatory elements and synteny analysis

*Cis*-regulatory elements (CREs) within the promoter region of *PvNF-Ys* genes were analyzed using sequences 1000 bp upstream of the start codon from Phytozome. The CREs of the *PvNF-Ys* genes were identified by PlantPAN 4.0 database (http://PlantPAN.itps.ncku.edu.tw/, accessed on 18 April 2024) [43]. For synteny analysis, genomes of *Panicum virgatum L.*, *Arabidopsis thaliana*, *Oryza sativa*, and *Zea mays* were downloaded from the Phytozome. MCScanX (Multiple Collinearity Scan Toolbox) was utilized with default settings to assess gene duplication events [44]. Dual syntenic maps were created by using TBtools to identify synteny relationships between paralogous *Panicum virgatum L*. genes and orthologous *PvNF-Ys* genes in *Arabidopsis*, *Oryza sativa*, *and Zea mays*.

### Functional annotation and transcriptome analysis of *PvNF-Ys* genes

To explore the functional annotation of the predicted *PvNF-Ys* genes, GO ontology analysis was carried out by using Eggnog (http://eggnog5.embl.de/ accessed on 22 April 2024) and visualized by WEGO 2.0 (https://wego.genomics.cn/ accessed on 22 April 2024). To investigate the expression level of all the identified *PvNF-Ys* genes, we obtained previously reported RNA-seq data from NCBI-GEO (https://www.ncbi.nlm.nih.gov/geo/, accessed on 2 May 2024) (accession no. GSE132772, and GSE174278) [45–47] for switchgrass subjected to drought and combination of drought and heat stress treatment.

### Plant growth, expression profiling under tissue/organ development and qRT-PCR analysis

To investigate expression profiling under tissue/organ development, data was retrieved from JGI database (https://phytozome-next.jgi.doe.gov/geneatlas/, 3 May 2024) and to validate the organ specific expression, switchgrass seedlings were grown at room temperature of 25 ± 1 °C, 16 h light/20 ± 1 °C, a total RNA was extracted from various part of six weeks old switchgrass plants including root, node, leaf blade, leaf sheath, shoot, and seeds using EasyPure Plant RNA Kit (Transgene Biotech, Beijing, China), and according to the manufacturer’s instructions, cDNA was synthesized through the EasyScript First-Strand cDNA Synthesis SuperMix (TransGen Biotech, Beijing, China). qRT-PCR was conducted by using SYBR Green Mix (Magic-bio, Hangzhou, China). The gene specific primers were designed by PrimerQuest™ Tool, and eEF1α was used as a reference gene. are shown in Table S4. The quantitave data was analyzed by 2−(^ΔΔCt^) method and excel was used for gene expression map.

## **Results**

### Identification of *PvNF-Y* genes in Switchgrass

Based on amino acid residues of NF-Y gene family of *Arabidopsis thaliana*, a total of 47 non-redundant protein sequences (17 PvNF-YA, 18 PvNF-YB, 12 PvNF-YC) representing primary transcripts were identified in switchgrass by using BLAST-P, after removing duplicates and incomplete sequences. According to their sub-family, structure and ID, genes were named as *PvNF-YA01* to *PvNF-YA17*, *PvNF-YB01* to *PvNF-YB18*, and *PvNF-YC01* to *PvNF-YC12* (Table S1). Further, we have conducted physiochemical properties analysis of all the predicted PvNF-Ys proteins. Results revealed that the protein length of all the 47 PvNF-Ys proteins ranged from 128 (PvNF-YC04, PvNF-YC12) to 376 (PvNF-YC08) residues (Table 1). The molecular weight ranged from 13550.4 (PvNF-YC12) to 40334.4 (PvNF-YC08) kDa, and the isoelectric point varies from 4.76 (PvNF-YC02) to 10.86 (PvNF-YA12). Further, the GRAVY index ranged from − 1.358 (PvNF-YC08) to -0.135 (PvNF-YC03), indicating that PvNF-Ys proteins were mainly hydrophilic, though the extent of their hydrophilicity varied (Table 1). A comparison of the three subfamilies of PvNF-Y exhibit distinct physiochemical properties. We found that *PvNF-YA* gene family is characterized by larger proteins with higher pI, which are generally associated with DNA binding and transcriptional activation, as NF-YA directly interacts with DNA in the NF-Y complex [16, 17]. In contrast, *PvNF-YB* and *PvNF-YC* subfamilies have lower molecular weight often act in specialized regulatory roles, interacting with other proteins to modulate gene expression [16].

**Table 1:** Physiochemical properties of all the predicted *PvNF-Ys* genes

Sub-family	Genes name	Gene ID	Protein length (AA)	Molecular weight (MV)	Theoretical (pI)
	*PvNF-YA01*	Pavir.2NG042700.1	256	27451.8	9.96
	*PvNF-YA02*	Pavir.2NG583300.1	292	31,777	8.44
	*PvNF-YA03*	Pavir.1KG112061.1	268	29206.9	10.76
	*PvNF-YA04*	Pavir.3KG543700.1	214	23366.6	7.98
	*PvNF-YA05*	Pavir.3KG551100.2	328	35,332	9.19
	*PvNF-YA06*	Pavir.9KG612800.1	240	26501.4	8.68
	*PvNF-YA07*	Pavir.9KG392700.5	314	34434.5	9.24
**NF-YA**	*PvNF-YA08*	Pavir.9KG040100.5	269	28460.6	9.39
	*PvNF-YA09*	Pavir.9KG069000.2	346	36702.2	9.51
	*PvNF-YA10*	Pavir.2KG064700.1	262	27864.6	10
	*PvNF-YA11*	Pavir.2KG529900.14	290	31,641	9.24
	*PvNF-YA12*	Pavir.1NG526800.1	267	29006.7	10.86
	*PvNF-YA13*	Pavir.9NG726300.1	239	26406.3	8.03
	*PvNF-YA14*	Pavir.9NG184100.1	346	36937.4	9.34
	*PvNF-YA15*	Pavir.9NG146600.2	267	28302.5	9.44
	*PvNF-YA16*	Pavir.3NG276300.1	331	35534.2	9.05
	*PvNF-YA17*	Pavir.3NG282900.1	214	23449.7	6.9
	*PvNF-YB01*	Pavir.2NG582200.1	217	22826.6	6.21
	*PvNF-YB02*	Pavir.6KG075900.1	252	25471.1	5.89
	*PvNF-YB03*	Pavir.4KG139640.1	245	26,541	9.09
	*PvNF-YB04*	Pavir.3KG485155.1	180	18951.1	6.31
	*PvNF-YB05*	Pavir.3KG314300.3	141	15493.2	5.17
	*PvNF-YB06*	Pavir.9KG391200.2	214	22,612	6.3
	*PvNF-YB07*	Pavir.2KG531100.1	217	22670.3	6.3
	*PvNF-YB08*	Pavir.5NG619400.1	159	17933.9	6.43
**NF-YB**	*PvNF-YB09*	Pavir.5NG619500.1	164	17466.4	6.2
	*PvNF-YB10*	Pavir.5NG576500.1	161	17649.8	5.96
	*PvNF-YB11*	Pavir.1NG467100.1	269	28294.3	6.59
	*PvNF-YB12*	Pavir.1NG409819.1	252	26847.7	6.35
	*PvNF-YB13*	Pavir.9NG528800.1	213	22601.9	6.3
	*PvNF-YB14*	Pavir.5KG753500.1	154	17250.2	6.43
	*PvNF-YB15*	Pavir.5KG607100.3	164	17846.9	5.82
	*PvNF-YB16*	Pavir.3NG079767.2	180	18,879	6.31
	*PvNF-YB17*	Pavir.3NG180531.2	138	15,105	5.3
	*PvNF-YB18*	Pavir.4NG231400.1	249	26821.2	9.57
	*PvNF-YC01*	Pavir.6NG263700.1	208	22254.5	5.48
	*PvNF-YC02*	Pavir.6NG097100.1	330	35403.6	4.76
	*PvNF-YC03*	Pavir.2NG380900.4	140	15427.6	5.36
	*PvNF-YC04*	Pavir.7NG445700.1	128	13568.4	6.83
	*PvNF-YC05*	Pavir.6KG104000.1	332	35965.2	5.2
**NF-YC**	*PvNF-YC06*	Pavir.6KG339000.1	208	22171.4	5.66
	*PvNF-YC07*	Pavir.1KG073400.2	259	28857.6	5.04
	*PvNF-YC08*	Pavir.9KG018900.1	376	40334.4	10.53
	*PvNF-YC09*	Pavir.1NG065700.5	260	29,147	5.05
	*PvNF-YC10*	Pavir.9NG568300.2	249	26153.4	5.2
	*PvNF-YC11*	Pavir.4NG275600.1	246	27417.1	5.13
	*PvNF-YC12*	Pavir.7KG370900.1	128	13550.4	6.83

### Multiple sequence alignment of PvNF-Ys proteins

**Fig. 1:**
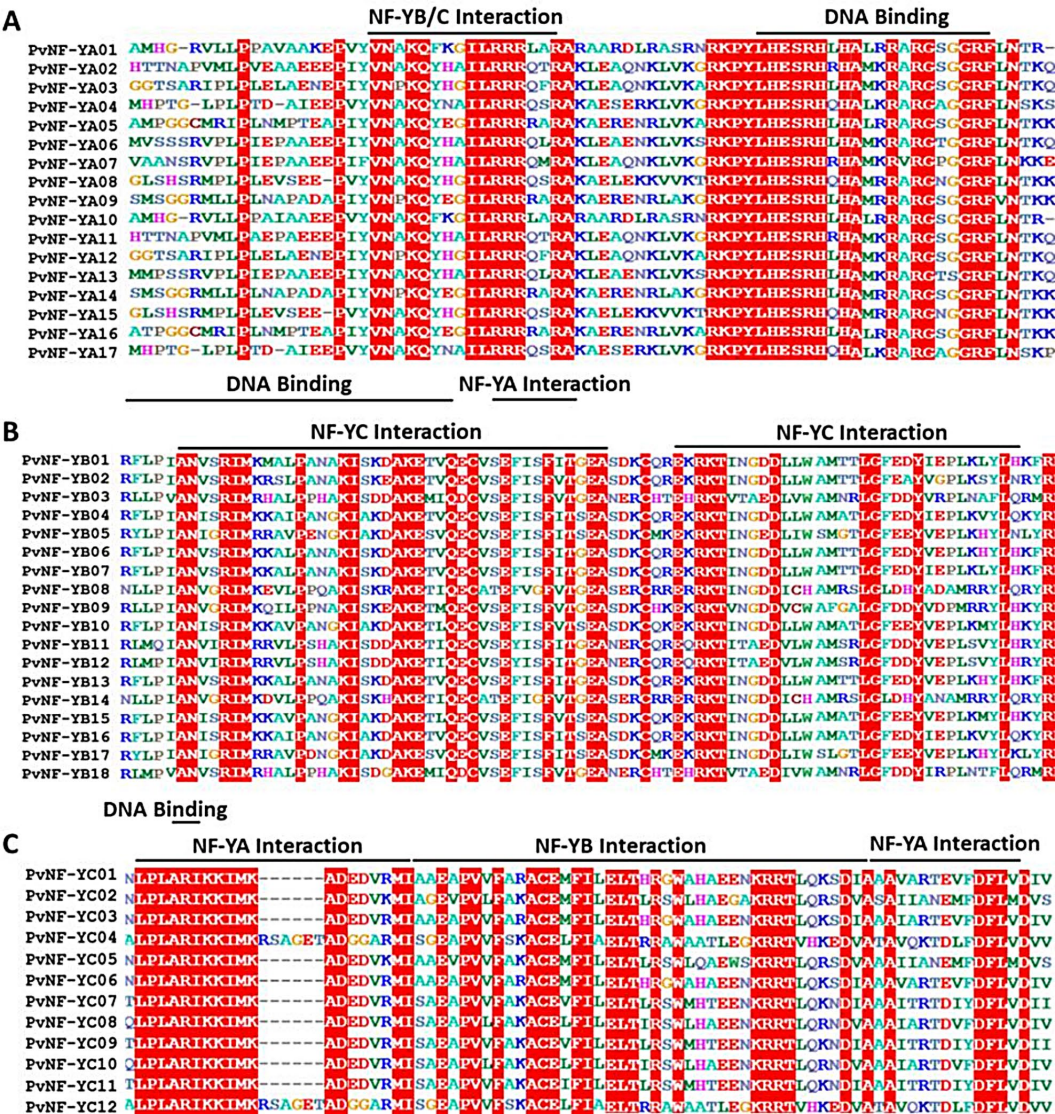
Multiple sequence alignment analysis of PvNF-Ys proteins. (**A**) PvNF-YA (**B**) PvNF-YB (**C**) PvNF-YC alignment of highly conserved domains in switchgrass

To identify and analyze the conserved domains, the sequences of PvNF-YA, PvNF-YB and PvNF-YC proteins were subjected to conduct multiple sequence alignment through ClustalW (Fig. 1). A highly conserved interaction and the DNA-binding domains were found in each member of these three subunit PvNF-Ys proteins. Each member of PvNF-YA contains DNA binding and NF-YB/C-interaction domain (Fig. 1A). Similarly, the PvNF-YB members have two NF-YC interaction domains separated by DNA binding and NF-YA domain (Fig. 1B). However, PvNF-YC members have two NF-YA interactions, one NF-YB interaction, and a two amino acid DNA-binding domain (Fig. 1C).

### Conserved motifs, phylogenetic and chromosome distribution analysis

**Fig. 2:**
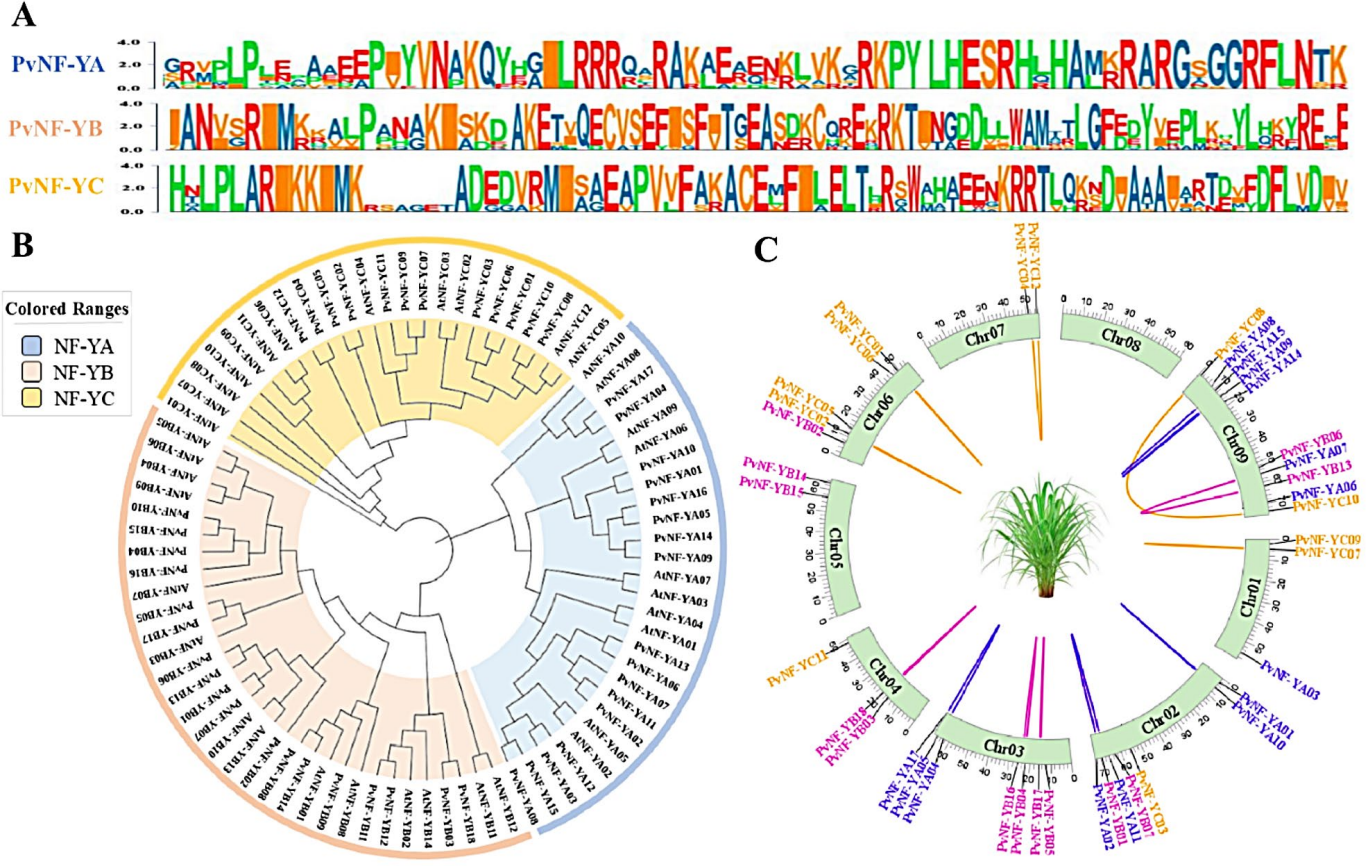
Conserved motif logos, phylogenetic tree, and chromosome distribution analysis in the *PvNF-Ys* genes. (**A**) A highly conserved sequence logos in the three subunits of PvNF-Ys proteins. (**B**) phylogenetic tree analysis of *PvNF-Ys* genes. (**C**) Chromosome distribution of *PvNF-Ys* genes are marked with different colors (NF-YA, blue; NF-YB, pink; NF-YC, orange)

A conserved motifs analysis was carried out through an online MEME program by using protein sequences of PvNF-Ys, and their highly conserved sequence logos are presented in (Fig. 2A). To further identify the evolutionary relationship between switchgrass and *Arabidopsis*, a phylogenetic tree through the neighbor-joining method was constructed by using 35 AtNF-Ys and 47 PvNF-Ys protein sequences. A total of 47 PvNF-Ys was divided into three subfamilies such as PvNF-YA, PvNF-YB and PvNF-YC based on the classification of *Arabidopsis* NF-Y gene families and the composition of conserved domains in the PvNF-Ys proteins. Among these subfamilies, PvNF-YC was smallest group with 12 genes. Whereas PvNF-YB had largest group with 18 genes. The PvNF-YA subfamily had 17 gene members (Fig. 2B).

We analyzed the genome of switchgrass to study the chromosomal distribution of *PvNF-Ys* genes. Our analysis showed that all the 47 *PvNF-Y* genes were unevenly distributed across 9 chromosomes. The maximum number of *PvNF-Ys* genes were found on chr9 (10), chr2 (7), chr3 (7) and chr6 (5). Chromosome 1 and 4 contained three *PvNF-Y* genes, while chr7 and chr5 each had only two *PvNF-Y* genes. however, no gene was found on chr8 (Fig. 2C).

**Fig. 3:**
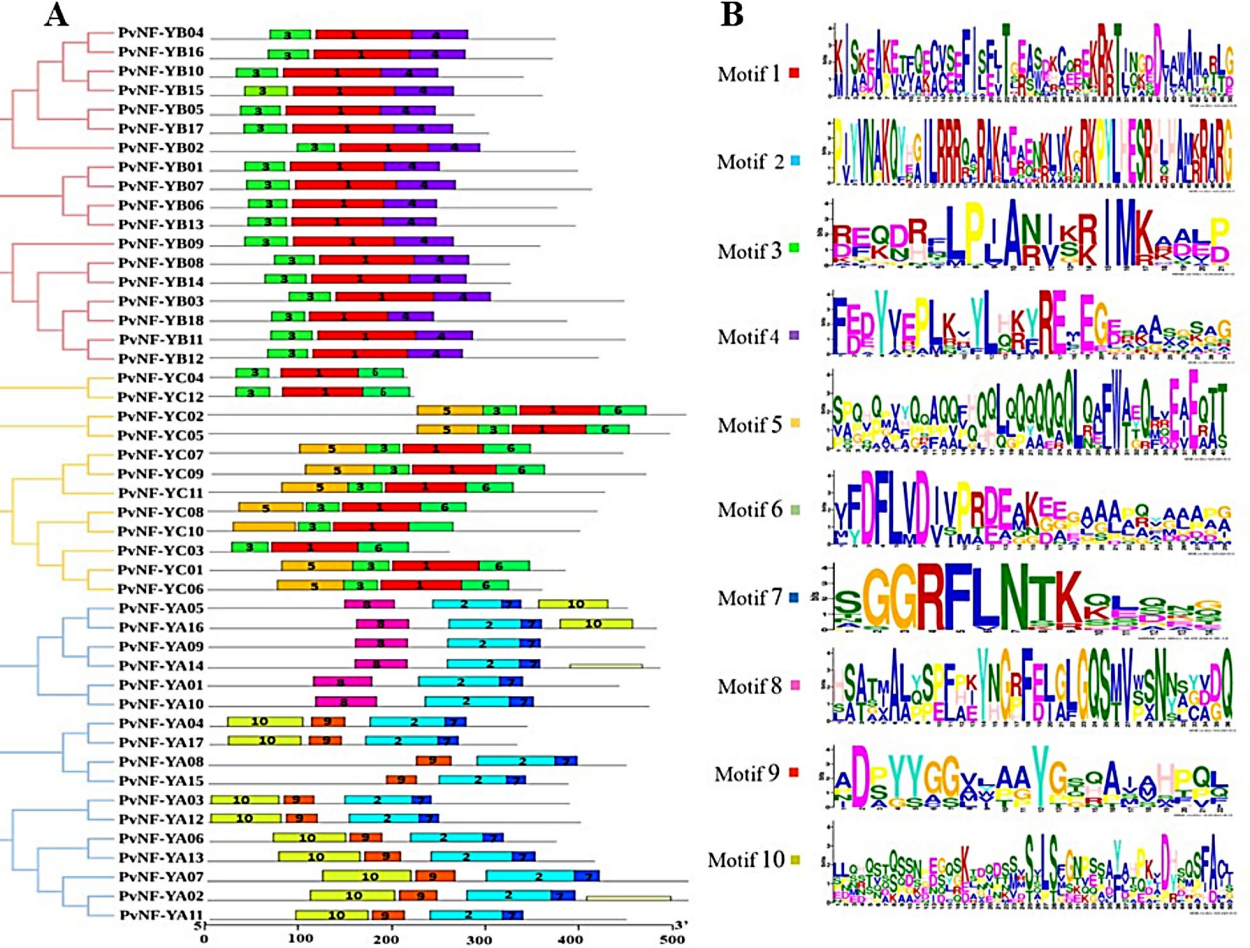
The distribution of conserved motifs in the *PvNF-Ys* genes. (**A**) Analysis of conserved motifs along with rectangular phylogenetic tree. Different color bars represented motif types in each subunit of PvNF-Ys proteins. The length of proteins can be estimated using the scale at the bottom. (**B**) Sequence information of 10 conserved motifs

Across the three subunit PvNF-Ys proteins, a total of ten motif logos with distinct amino acid sequences were identified. In the PvNF-YA subunit members, motifs 2,7,8,9 and 10 were detected, with motifs 2 and 7 found consistently across all members of the PvNF-YA subfamily. In the PvNF-YB subfamily, all the members shared similar distribution of motifs including 1,3 and 4 motifs. However, motifs 1 and 3 were also detected in the PvNF-YC subfamily members along with motif 5 and 6 (Fig. 3A). All the ten-motif logos were displayed in (Fig. 3B).

### Functional domain and gene structure analysis of *PvNF-Ys* genes

**Fig. 4:**
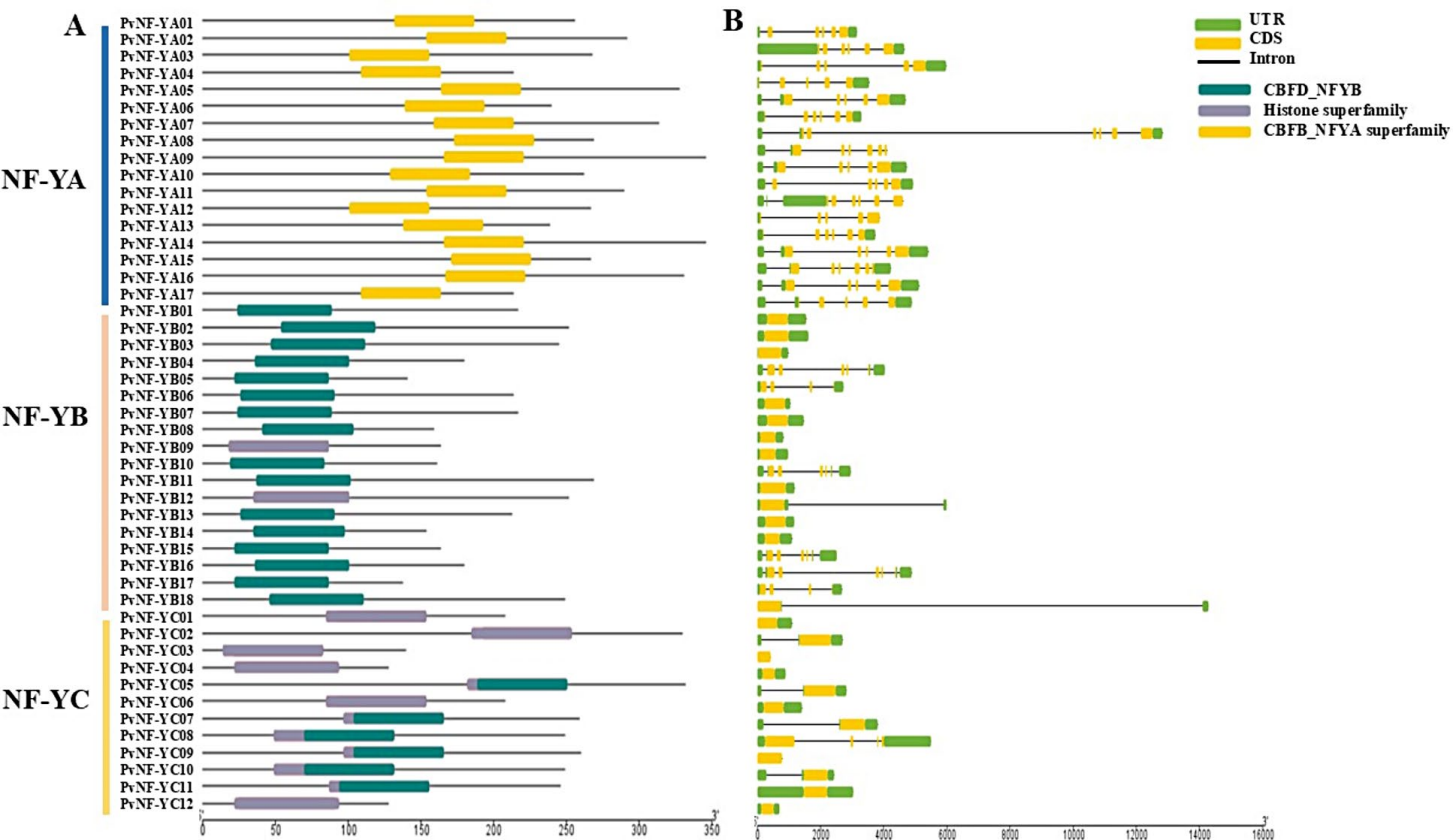
Distribution of domains and gene structure prediction of *PvNF-Ys* genes in switchgrass. (**A**) Conserved domains of *PvNF-Ys* genes. Different colour boxes represent the conserved domains. (**B**) The number of introns, CDS and UTR were represented in black lines, yellow bars, and green bars in each gene

The conserved domain distribution was identified by NCBI conserved domain database. Analysis results revealed that members of PvNF-YA family contain conserved domain CBFB-NFYA (CCAAT-binding transcription factor B-NFYA), or the CBFB-NFYA superfamily (Fig. 4A). PvNF-YB and PvNF-YC families possess conserved regions within the CBFD-NFYB-HMF (CCAAT-binding transcription factor D-NFYB histone-like transcription factor) family or Histone superfamily (Fig. 4A). Additionally, to improve our understanding of gene architecture, the genomic DNA sequences of the *PvNF-Y* genes were analyzed to assess and compare the structures and counts of exons, introns, and UTRs. The results showed that majority of *PvNF-YB* and *PvNF-YC* genes had either no or only a few introns. Moreover, each *PvNF-YA* gene have maximum number of introns and exons. The number of UTRs were found in *PvNF-Ys* ranging from 1 to 4. However, there was no UTR found in *PvNF-YC03* and *PvNF-YC09* genes (Fig. 4B).

### Orthologous gene pairs among switchgrass and various plant species

**Fig. 5:**
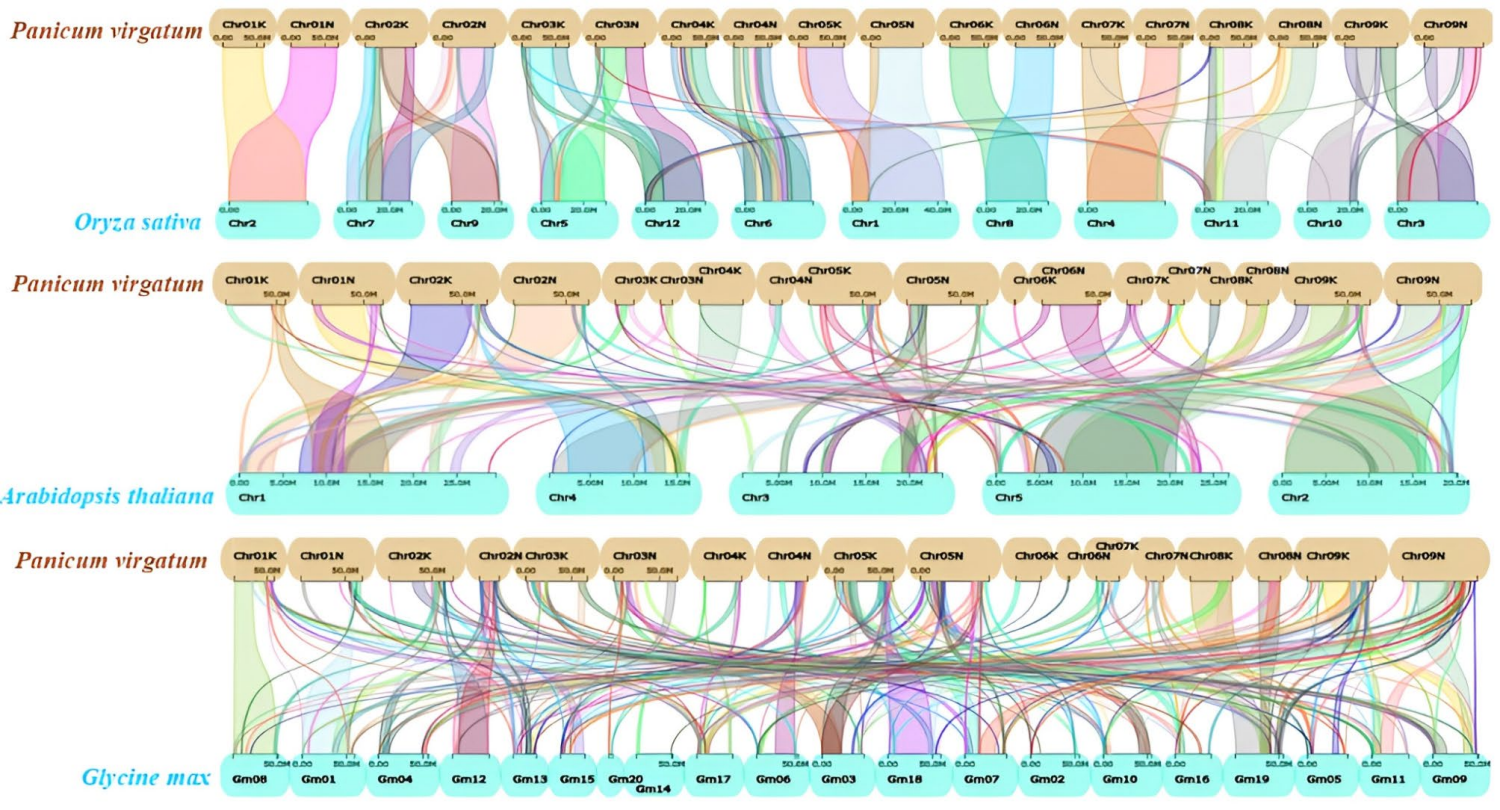
Dual synteny analysis of switchgrass *PvNF-Ys* genes with dicot and monocot plant. Yellow bars represent chromosomes of switchgrass, while blue bars represent chromosomes of *Arabidopsis*, *Oryza sativa* and *Glycine max*. Colour lines emphasize syntenic *PvNF-Ys* gene pairs within the genomes of switchgrass and other plants. Dispute lines denote collinear blocks, while fine lines in the background emphasize syntenic *NF-Y* gene pairs within the genomes of switchgrass and other plants

The orthologous gene pairs offer insights into the evolutionary relationships among different plant species. To gain a deeper understanding of the evolutionary connections of *PvNF-Ys* genes in dicot and monocot plants, a dual synteny analysis was performed between switchgrass (*Panicum virgatum L.*), *Arabidopsis*, *Oryza sativa* and *Glycine max.* These results indicate that collinear relationship between switchgrass, *Glycine max* and *Arabidopsis thaliana* was most significant while between *Oryza sativa* was less prominent (Fig. 5). These results provide clues for examining the relationship between functional genes. Beyond that, these findings can help to demonstrate the homologous feature in the genome organization of different plants and further explore the way of functional evolution of *NF-Y* gene pairs in the process of plant evolution.

### Subcellular localization, functional annotation and *cis*-regulatory elements analysis

**Fig. 6:**
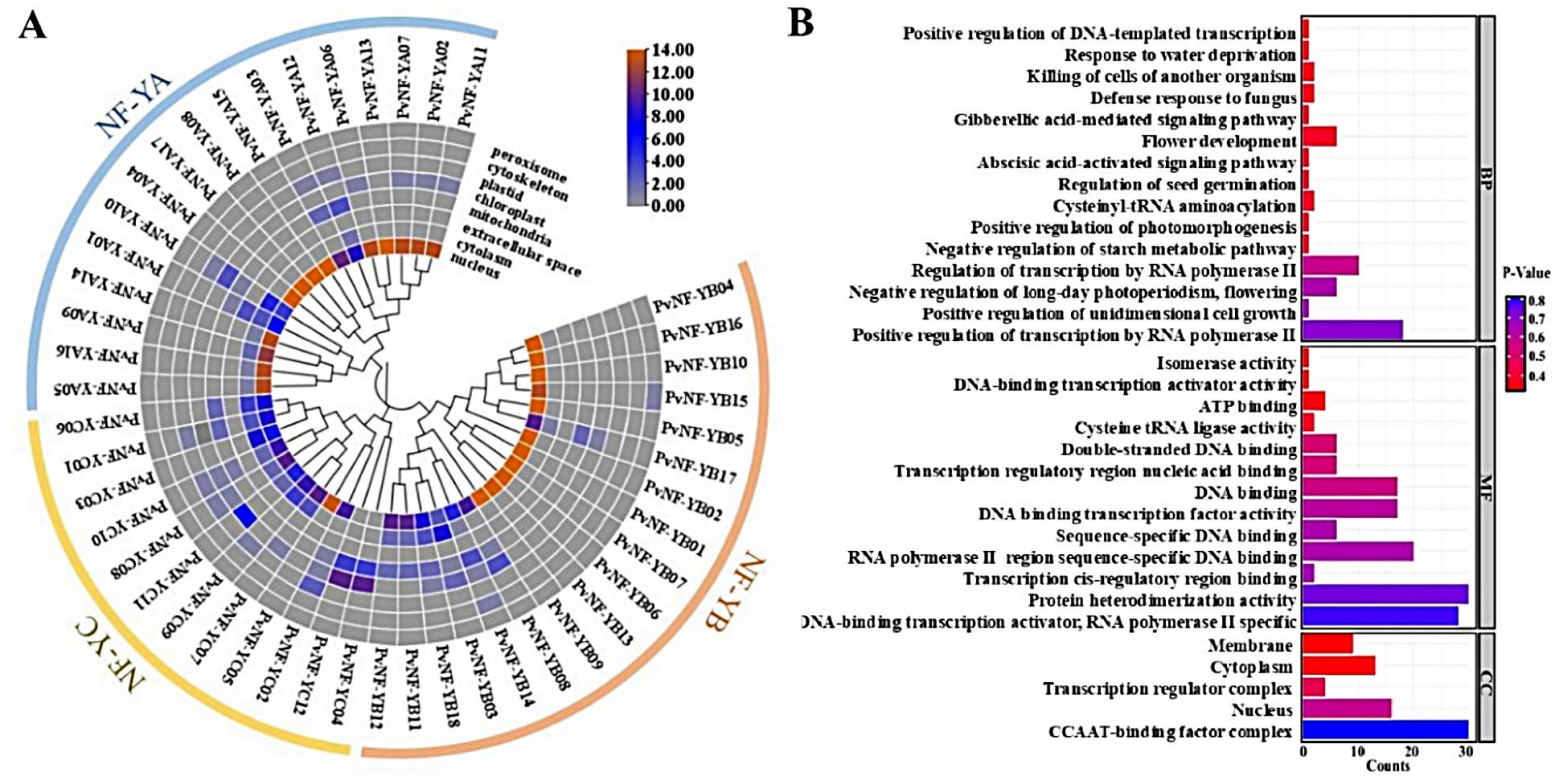
In silico subcellular localization and functional annotation analysis of all the predicted PvNF-Ys proteins. (**A**) Subcellular localization analysis in the PvNF-Y proteins, side bar shows highest count present in each category. (**B**) Gene ontology analysis of the predicted *PvNF-Ys* genes significantly enriches < 1 P-value

To determine the subcellular location of PvNF-Ys proteins, we performed an in silico sub-cellular localization analysis using WOLF PSORT. The results showed that most of the predicted PvNF-Ys proteins were predominantly located in the nucleus except the PvNF-YC04 and PvNF-YC12, were found in the mitochondria (Fig. 6A). To further analyze functional annotation, all the identified 47 *PvNF-Ys* genes were successfully annotated for their potential functions using Eggnog. The Gene ontology (GO) analysis results showed that *PvNF-Ys* genes were characterized into three categories; molecular function, biological process and cellular components. The *PvNF-Ys* genes were significantly involved in many biological processes including positive regulation of transcription by RNA polymerase II, negative regulation of long-day photoperiodism, flower development and positive regulation of unidimensional cell growth. Further, *PvNF-Ys* genes were predicted to be more frequently involved in many molecular functions such as RNA polymerase II-specific, protein heterodimerization activity, *cis*-regulatory region sequence-specific DNA binding, and DNA-binding transcription factor activity. Furthermore, *PvNF-Ys* were involved significantly in the CCAAT-binding factor complex, nucleus, and cytoplasm as illustrated in cellular component category (Fig. 6B).

**Fig. 7:**
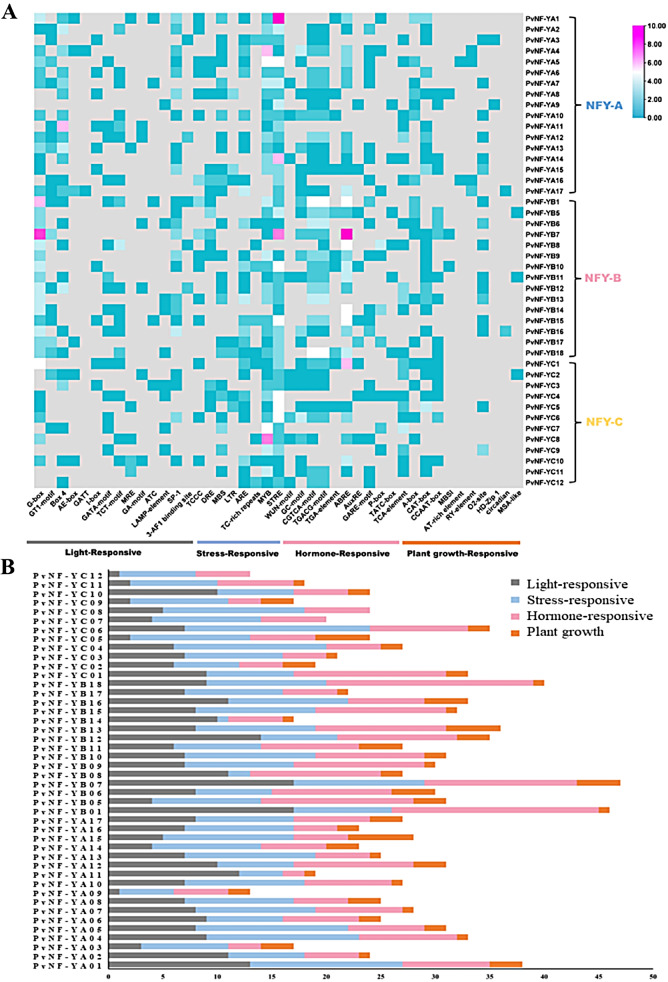
In-silico *cis*-regulatory elements analysis of the predicted *PvNF-Ys* genes. (**A**) *Cis*-regulatory elements motifs analysis in their respective groups were associated with different phytohormones, responses to stress and light, growth and developmental processes. (**B**) Different color bars represent the highest count based on the number of times a particular *cis*-element occurs within the promoter in each member of *PvNF-Y* gene family

*Cis*-regulatory elements are DNA-binding motifs located in the promoter regions of genes that regulate transcription. In silico analysis of *cis*-regulatory elements can be performed to evaluate the potential functions of various genes. In *PvNF-Ys* genes many *cis*-regulatory elements were detected at the promoter region. Several phytohormone and stress-related motifs including DRE, MBS, LTR, ARE, TC-rich repeaters, MYB, STRE, WUN-motif, GC-motif, CGTCA-motif, TGACG-motif, ABRE (Abscisic acid-responsive element), GARE-motif, P-box, TATC-box (Gibberellin-responsive element), AuxRE, TGA-element (Auxin-responsive element) and TCA-element (Salicylic acid-responsive element) were detected in the promoter region, suggesting that the expression of *PvNF-Ys* genes may be regulated by multiple phytohormone. Moreover, stress-related *cis*-regulatory elements motifs findings indicated that *PvNF-Ys* genes may be closely associated with multiple biotic and abiotic stress responses. Furthermore, many motifs related to plant growth, development and other elements such as A-box, CAT-box, CCAAT-box, MBSI, AT-rich element, RY-element, O2-site, HD-Zip 1, Circadian and MSA-like were found in *PvNF-Ys* genes. Several light responsive such as G-box, GT1-motif, Box 4, AE-box, GATT, I-box, GATA-motif, TCT-motif, MRE, GA-motif, ATC, LAMP-element, SP-1, 3-AF1 binding site, and TCCC were detected in the *PvNF-Ys* genes (Fig. 7A, Table S2). Overall, statistics of *cis*-regulatory elements in the *PvNF-Y* gene family were calculated. All the predicted *cis*-regulatory elements are categorized by different colors based on their association with light, stress, hormone and plant growth (Fig. 7B). The results indicated that stress-responsive and hormone-related motifs were significantly dominant among the predicted *PvNF-Ys* genes (Fig. 7B, Table S3).

### Expression patterns of *PvNF-Ys* genes under drought and heat stress

**Fig. 8:**
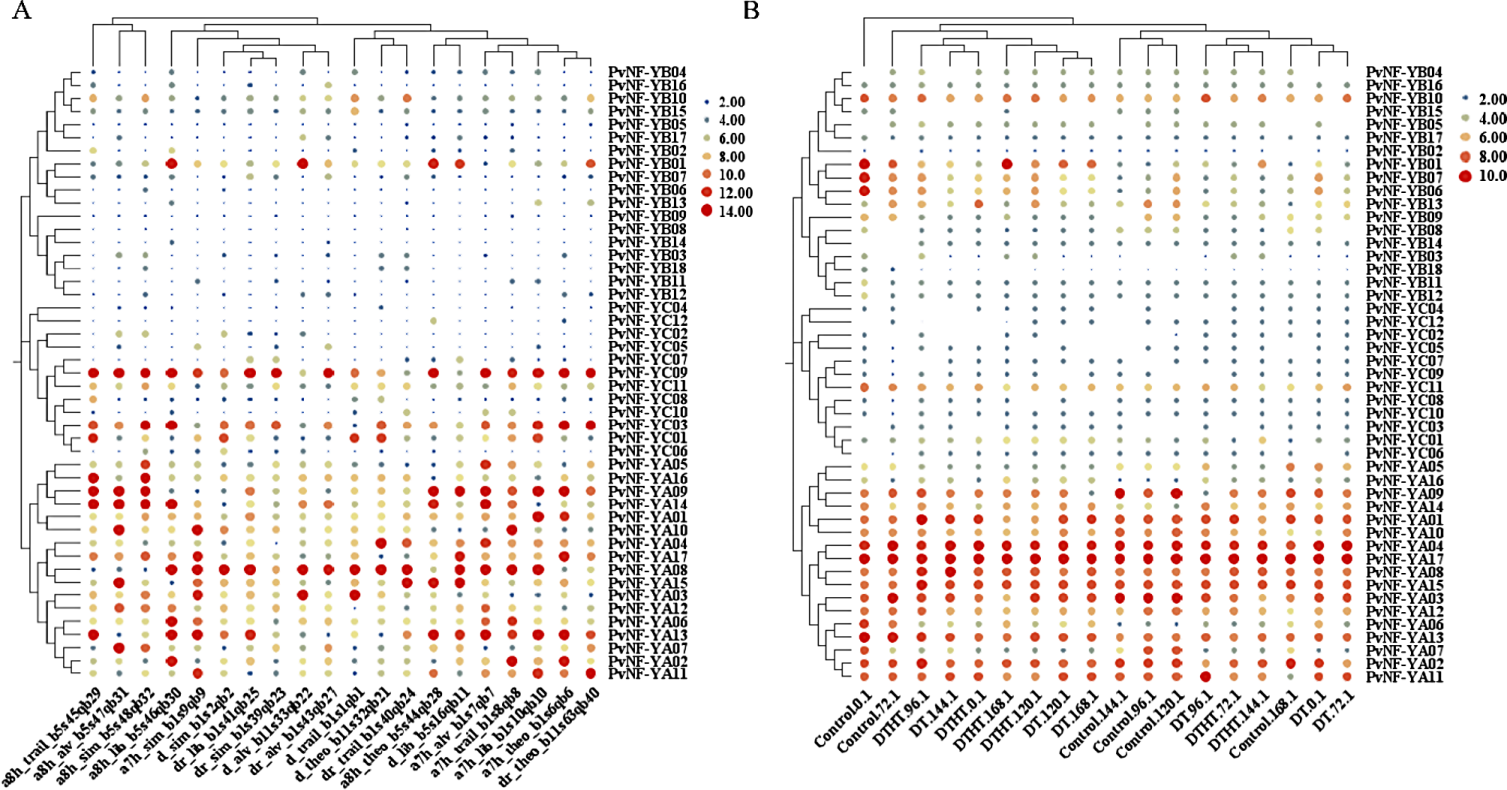
The expression profile of *PvNF-Ys* genes under drought stress treatment. (**A**) The colors bar represents level of expression based on FPKM values: orange/high expression, yellow/low expression, blue/no expression. (**B**) The expression profile of *PvNF-Ys* genes under drought and combined drought and heat stress depend on FPKM values: green/high expression and Yellow/low expression

To explore the potential role of *PvNF-Ys* genes under drought stress and plant development. Here, we compiled the expression pattern of *PvNF-Ys* genes by using data from NCBI-GEO. The experiment (accession no; GSE132772) was conducted on gene expression and physiological responses in switchgrass under drought stress [47]. This study led to understanding the effects of whole genome duplication and water stress on growth, physiology, and gene expression. Tetraploid liberty and its neo-octoploid cultivars were subjected to drought and recovery conditions and mRNA sequencing of collected samples was performed using Illumina. The expression pattern of all *PvNF-Ys* genes was retrieved from this dataset. Among the 47 *PvNF-Ys* genes, most of the *PvNF-YA* genes exhibited high expression patterns compared to *PvNF-YB* and *PvNF-YC* gene families (Fig. 8A).

Another RNA-Seq study (accession no; GSE174278) investigated the transcriptomic response of switchgrass (*Panicum virgatum L.*), genotype Alamo AP13, to drought and combined drought and heat stress [45, 46]. The experiment involved drought treatment alone and a combination of drought with heat stress (35 °C/25°Cday/night). Samples were collected at multiple time points (0, 72, 96, 120, 144, and 168 h). The expression profile of all *PvNF-Ys* genes was re-analyzed from this dataset, we found that most of the *PvNF-YA* genes showed high expression level compared to *PvNF-YB* and *PvNF-YC* family’s members under different drought stress levels (Fig. 8B).

### qRT-PCR analysis of specific tissue/organ development for the *PvNF-Ys* gene family

**Fig. 9:**
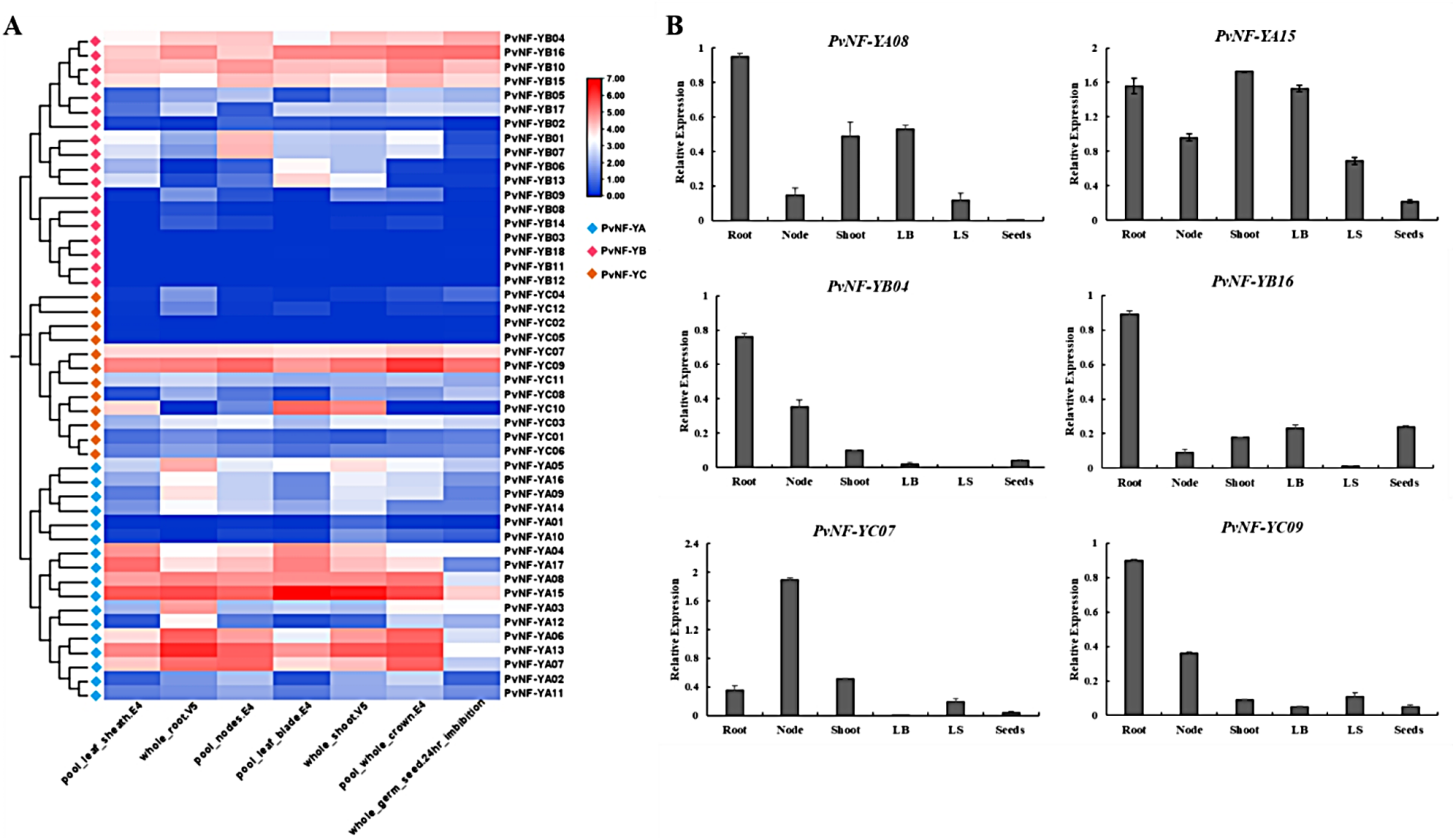
Expression analysis of specific tissue/organs of the *PvNF-Ys* genes. (**A**) Expression profiling of *PvNF-Ys* in tissue and organ development using data through JGI database (**B**) Quantitative-real-time PCR showed expression of genes in different organs

To further investigate the potential role of *PvNF-Ys* genes in plant growth and development stages, we conducted expression profiling of *PvNF-Ys* genes across various tissue/organ using data from the JGI database. The results showed that most of the *PvNF-Ys* genes were expressed in most tissue/organ, but the level of expression varied (Fig. 9A). To validate the organ/tissue specific expression profile of *PvNF-Ys* genes, we carried out qRT-PCR experiment utilizing gene-specific primers. Several *PvNF-Ys* genes were selected based on evolutionary relationship between them, we found that almost all the selected *PvNF-Ys* genes except *PvNF-YC07* shown high expression level in root compared to other organs (Fig. 9B).

## Discussion

NF-Ys factor is essential for plant development and proliferation at different stages. Abiotic stressors like salt, drought, and temperature significantly impact plant development, leading to adaptation mechanisms for root growth, plant-microbe interactions, and water stress response [48]. *NF-Y* genes have garnered a lot of interest in various areas of agricultural research. In this study, we found 47 *PvNF-Ys* genes, with 17 *PvNF-YA*, 18 *PvNF-YB* and 12 *PvNF-YC* (Table 1). However, number of genes varies over different species. A total of 24 *PpNF-Ys* were found in *Prunus persica* [19] with 6 *PpNF-YAs*, 12 *PpNF-YBs*, and 6 *PpNF-YCs*, while in *Petunia hybrida* have 27 *PhNF-Ys* with 10 *PhNF-YAs*, 13 *PhNF-YBs* and 12 *PvNF-YCs* [49].

A phylogenetic study revealed a strong relationship between switchgrass (*Panicum virgatum L.*) *PvNF-Y* genes and *Arabidopsis AtNF-Y* genes. All the 47 identified *PvNF-Ys* genes were categorized into three subgroups corresponding to *AtNF-Y* genes (Fig. 2B). That is similar to the NF-Ys discovered in *Arachis hypogaea* [19], *Prunus persica L* [49], and *Solanum tuberosum L* [50]. The roles of *PvNF-Y* genes can be deduced from the phylogenetic tree’s *Arabidopsis* genes with known functions. The *PvNF-YB2*,* B11*,* B17*, and *B05* share lineage with *AtNF-YB2* and *B3* specifying their role in the regulation of flowering time [23]. Additionally, *AtNF-YC4* makes clade with *PvNF-YC7*,* C09* and *C11* plays significant role in blooming and photomorphogenesis [4]. Moreover, *PvNF-YB03*,* B11*,* B12*, and *B18* contained aspartic acid residue at position 55 indicating that they may be LEC1 type genes while others are non-LEC type [52]. In *Arabidopsis thaliana AtNF-YA2* and *A10* genes were seen to be *highly e*xpressed in roots. In the present case, according to qRT-PCR screening expression levels of selected *PvNF-Ys* genes are elevated in root tissue. Sideways, *PvNF-Ys* genes have a greater number of stress-responsive elements in promoter sequences. This leads to the conformation of their stress-resilient capabilities [5].

Multiple sequence alignments (Fig. 1) have shown that the PvNF-YA, PvNF-YB, and PvNF-YC subfamilies are characterized by DNA-binding domains that bind to specific CCAAT binding sites [51]. A sequence of 21 amino acids is displayed in the conserved region at the C-terminal of PvNF-YA: Y-L-H-E-S-R-H-x-H-A-x-x-R-x-R-G-x-G-G-R-F. This sequence, which comes from the DNA binding domain of PvNF-YA present in plants, mammals, and yeasts, is assumed to be linked to DNA at CCAAT locations. PvNF YA’s conserved area’s N-terminal region has the sequence Y-V-N-A-K-Q-x-x-x-I-L-R-R-R-x-x-R-A-K-L-E. The structure and amino acid composition of the conserved areas of NF-YB were comparable to those of H2B motifs. Based on the equivalent portions of NF-YB members in *Arabidopsis*, the 31-amino acid sequence R-L-P-x-I-A-N-x-x-R-I-M-x-x-x-x-P-x-x-x-K-I-x-x-x-A-K-E-T-x-Q was identified as the DNA-binding domain of PvNF-YB [52]. During the alignment examination of 12 PvNF-YC members, the interaction domain of *Arabidopsis* revealed similarities with a conserved 74 amino-acid sequence L-P-L-A-R-I-K-K-I-M-K-x-x-x-x-A-D-x-x-V. This led to the conclusion that PvNF-YC related to PvNF-YA/PvNF-YB through this fragment, which served as its centre area. Furthermore, just two critical residues from series “A” and “R” were present in PvNF-YC’s DNA-binding domain, which was necessary for heterotrimeric NF-Y and DNA to form a complete structure as reported in previous study [53].

In addition, motifs analysis (Fig. 3A) revealed only three (motifs 1, 3, and 4) were substantially shared by all PvNF-YA, PvNF-YBs have motifs 1, 3, 5, and 6, except for PvNF-Y04 and PvNF-YB12, which do not have motif 5. PvNF-YCs have a lot of motifs 2, 5, 8, 9, and 10. Similar to the previous research on peach [19]. Gene structure analysis, 27 PvNF-Y sequences had introns, whereas one-third of PvNF-Y sequences had single exons and zero introns (Fig. 4B). The results aligned with earlier studies that demonstrated that short intron length or absence increased plant gene expression [53, 54]. According to earlier research, the NF-Y members’ gene structure was strongly correlated with their evolutionary relationships [55–57]. Research on other plants has revealed that whereas NF-YBs and NF-YCs are more varied, the majority of NF-YAs have three to six introns in their genome sequence [22, 58, 59]. Additionally, a number of NF-YB and NF-YC members have been discovered without any intron like in *chickpea* [60], *S. bicolor* [55], *Ricinus cummunis* [61].

The location of genes are very important to determine their biological activity in the cells [62]. The majority of genes are located in the nucleus indicating its role in gene expression, with a smaller proportion being found in the cytoplasm and other organelles. Numerous *cis*-acting elements linked to plant growth and development as well as stress response were also found during the examination of the *panicum virgatum NF-Y* gene families. The CREs results indicated that stress-responsive and hormone-related motifs were significantly dominant among the predicted 47 *PvNF-Ys*. Phytohormones play a significant role in plant growth and development [63]. Light and plant growth responses were also found (Fig. 7B).

The expression profiles of every *PvNF-Ys* gene under drought stress [47] were examined in this work. *PvNF-YAs* exhibited high resilience to circumstances of drought and recovery (Fig. 8A). Further examination of drought and heat stress was conducted [45, 46]. In contrast to the *PvNF-YB* and *PvNF-YC* gene families, the majority of the *PvNF-YA* genes showed strong expression patterns (Fig. 8AB). The *PvNF-YA* members show elevated expression level during stress maybe because of its critical involvement in the transcriptional regulation associated with stress tolerance and having high number of stress-responsive elements in promoter regions. Recent studies have demonstrated that NF-YA subunits can activate or modulate genes related to water retention, osmotic adjustment, and efficient nutrient mobilization, which are essential under drought stress [64].

Gene ontology results (Fig. 6B) showed that these genes were significantly involved in regulating transcription by RNA polymerase II, regulation of cell growth, and unidimensional cell growth processes, while protein heterodimerization activity and DNA-binding transcription activator activities were most observed molecular functions of NFY transcription factor which are characteristic of NF-Y members and are found in a wide range of organisms, from plants to mammals [65]. In results, CAAT-binding factor complex found as a most significant cellular function [59]. Tissue/organ specific expression profiling results revealed that most of the *PvNF-Ys* genes were expressed in different organs, but the level of expression differed (Fig. 9A). Furthermore, we conduct qRT-PCR experiment (Fig. 9B) to verify the expression levels of particular *PvNF-Ys* genes in different organs by using gene-specific primers [66]. These findings are crucial for identifying potential genes that increase molecular breeding’s efficiency. Also, our results help choose *Panicum virgatum* L. cultivars with strong resilience to adversity and can be a vital resource for understanding how NF-Y transcription factors function in stressful conditions.

## Conclusions

The complete analysis screened 47 *PvNF-Ys* genes in switchgrass (*Panicum virgatum* L.), and is divided into three subfamilies (NF-YA, NF-YB, NF-YC). This study revealed the maximum number of NF-Ys residues on the ninth chromosome. Multiple sequence alignment exhibits DNA binding along with interacting domains. Phylogenetic analysis revealed the evolutionary relation of *PvNF-Ys* and *AtNF-Ys*. Gene structure showed one-third of *PvNF-Ys* lack intron. *Cis*-regulatory elements associated with stress-responsive and hormone-responsive elements motifs in the promoter region. Previously reported RNA seq data suggested that *PvNF-YAs* gene family showed high expression towards drought, combination of drought and heat stress compare to *PvNF-YBs* and *PvNF-YCs* gene family. qRT-PCR analysis showed that *PvNF-YA08*, *PvNF-YB04*, *PvNF-YB16*, and *PvNF-YC09* highly expressed in roots, (except *PvNF-YC07* gene). These findings deepen our understanding of how *PvNF-Y* genes play a role in cellular processes and environmental responses, with implications for crop resilience and biotechnology applications.

## Electronic supplementary material

Below is the link to the electronic supplementary material.

Supplementary Material 1

Supplementary Material 2

## Data Availability

All the data generated or analyzed during this study are included in this published article and its supplementary data files. The genome file of switchgrass (*Panicum virgatum* L.) was downloaded from Phytozome v13.0 (https://phytozome-next.jgi.doe.gov/). The Protein sequences of NFYs transcription factor gene families of *Arabidopsis thaliana* were obtained from TAIR (https://www.arabidopsis.org/browse/genefamily/index.jsp).
