# Supplementary material for: Genome-wide analysis and identification of nuclear factor Y gene family in switchgrass (Panicum virgatum L.)
Source: BMC Genomics. 2024 Dec 20;25:1218. doi: 10.1186/s12864-024-11092-6 (PMC11661050; doi:10.1186/s12864-024-11092-6)
Supplement: Supplementary file 2 — Supplementary Material 2 [file 12864_2024_11092_MOESM2_ESM.pdf]

File S1

Protein sequences of PvNF-Ys and AtNF-Ys

>PvNF-YA01

MRSAAAMCFQEHGLAFQVVADGGRGHGGGATAAPWWATTAQGFAAKFPISFSSGDSDPPQDHKYHEPSAAGMAAYPELHKYHGPFDLAFGQSMVWSNNAGAGQRQSYGLYSPY
GAQAMHGRVLLPPAVAAKEPVYVNAKQFKGILRRRLARARAARDLRASRNRKPYLHESRHLHALRRARGSGGRFLNTRSLAAGDPAPPGGPEPTEDSASKAATLHPVQDRQDVFH
SSLTNMAGDGDGRWACAAPRGCRDLLKA

>PvNF-YA02

MPKLLREVEDHPVHPMSKSNHGFLSGNGHEMKHLGHKIHDKDSSSESGQSHQEAPAVSESSLNENTSTQSDNDEGHGKHNQDTEHSVLSMGKQGSAFLPPKLDYNPSFACLPYTAD
AYYGGVLPGYPPHAIVHPQQNHTTNAPVMLPVEAAEEEPIYVNAKQYHAILRRRQTRAKLEAQNKLVKGRKPYLHESRHRHAMKRARGSGGRFLNTKQLQEQSQQHQASGGSSSS
KFIGNSISSQSDPTPTPSTPASSDTASASRVNQDHNCFPSVGFRPAMNFSAPGGDGAKLVS

>PvNF-YA03

MLLPSSSSSSASKGNSLRKTVIDNMRSTLSFENKQPLFASQAIDYGQPIACISYPYNDSGSGGVWAAYGSRTSSSTVFHPQIAGGGTSARIPLPLELAENEPIYVNPKQYHGILRRRQFRAK
LEAQNKLVKARKPYLHESRHLHAMKRARGSGGRFLNTKQLQQQPRNASTRSTTNGTASSSGSTHLRLGGGTARDQTMLAPKAMPSQYNSKKAVSSAPGFTVTPMLHRDDTFFQHPS
HHLSFSGHFGQASAQAGMHNGTQQRVPVMR

>PvNF-YA04

MTSVVHSVSGDHRAEDQHQQQKQAVPEDQQEAPVTSSDSQPTVGTPSADYVAPYAPHDMGHALGQYAYPNIDPYYGSLYAAYGGQPMMHPPLVGMHPTGLPLPTDAIEEPVYVNA
KQYNAILRRRQSRAKAESERKLVKGRKPYLHESRHQHALKRARGAGGRFLNSKSDDKEENSDSSHKEKQNGVAPHKSGQPSTPPSPNGASSANQADSRQ

>PvNF-YA05

MMSFRSHEGFGQVPAGATSNGAAVPWWAPAPQLLLYGDALGQGKVPPEQAPCREARFQVVPGAQAPLGPPVQPPAKAAAERGLPVLKFSVAQGKGEKGSEHSASVALPSPFAIYNG
RFELGLGQSTVSVNSPYVDQHCGLISSYPVGAMPGGCMRIPLNMPTEAPIYVNAKQYEGILRRRRARAKAERENRLVKARKPYLHESRHLHALRRARGSGGRFLNTKKESNTKDAGG
DGKAMFSNPLMRQVASPSSEIQQSDLGNPSSVSSLSGSEVSSIYDHEDVDHFHSFDHIRTPFFTPLPSIMDSEHGVGNPFKWPIASEGCCDLLRA

>PvNF-YA06

MLPPHLTENGTIMIQFGHQMPDYNSPATQSTSESHQEVSGMSEGSLNDHNDQSGNHDGYSKSDENKMMSALSLGNPETAYAHPKTDRSQSFAISYPYADSYYGGAVAAYGQHAIQM
HPQLVSMVSSSRVPLPIEPAAEEPIYVNAKQYHAILRRRQLRAKLEAENKLVKSRKPYLHESRHLHAMKRARGTGGRFLNTKQQSDSPGGTSDAQRMPANGGLFTKHEHSLPPGDRH
YHARGGV

>PvNF-YA07

MMPSGNKMLLQEMDGDPFHTMQNYDFLSGNGYSMKQLIHSNSDKDSSSTKSEQSRQDLSTVSDSSLNGQHTPTQSDNNDSCGKRDQGMVKTILSFGNPEVAFSTQKFDHSQPFACA
SYTADPYYCGALTGYTSNAIVHPQINVAANSRVPLPIEPAAEEPIFVNAKQYHAILRRRQMRAKLEAQNKLVKGRKPYLHESRHRHAMKRVRGPGGRFLNKKELQEQQQQKALPSLQ
TPTGGAGKMALCRNNLCSENSASHSSSTSSGISIVSNGGGMVAHHEHISFSSTSFLPGMNFHAENGGEKMAVHGVHRRTPS

>PvNF-YA08

MESRPGGTNLVEPRGQGAALPSGGAAMQKPWWTSSGAGLGAVSPAVVAPGSGAGISLSSSPVGGGGGGGAGAAKGAASDESSEDSRRSGEPKDGSAGQEKNHATSQMPALMSEYL
TPYSQLELNQSIGSAAYQYPDPYYAGMIPPYGTQAVAHFQLPGLSHSRMPLPLEVSEEPVYVNAKQYHGILRRRQSRAKAELEKKVVKTRKPYLHESRHQHAMRRARGNGGRFLNTK
KSDNGTPNGNAEPKKGDQNYEHLHVPPDLLQLRQNEA

>PvNF-YA09

MMSFKGHEGLGQVAAGSQASHVAAGAPLPWWAGPQLIYGEPAPLSPEETRRDGHFQVVPGAQGGTPDPAPPAATKRGNPEVLKFSVFQGNLESGGKGDKNPEHSTTIALQSPFPEY
NGRFEIGLGQSMVPSNYSCDDQCYGVLTTYGMRSMSGGRMLLPLNAPADAPIYVNPKQYEGILRRRRARAKAERENRLAKGRKPYLHESRHLHAMRRARGSGGRFVNTKKEGGAS
ANGASKTVSAAPAARLATSPSTEPRRVPGLGKGSDGSNPRCQSRSSVSSLSGSEVSSIYDDHASRHQYGGMEPHLRAPSFFTPLPAIMDGDHGGAAAIPSFKWAASDGCCELLKA

>PvNF-YA10

MRSAAAMGFQEHGLAFQVITDGGGGATAAPWWAAAAAPQGFAAKFPIISSGDSDPPQDLYHEPLAAAMAAYPELHKYHGPFDLALGQSMVWSNNAGAGQRQSFGLYSPYGAQA
MHGRVLLPPAIAAEEPVYVNAKQFKGILRRRLARARAARDLRASRNRKPYLHESRHLHALRRARGSGGRFLNTRSLAAGDPPPQLASTPLGGPEPTEGGASKAARLHPAKERQEDVF
LSSLTNMAGDGDGKARWVGAAPRGCCDLLRA

>PvNF-YA11

MPKLLREVEDHPVHPMSKSNHGFLSGNDHEMKHLGHKIHDKDLSSESGQSHQEAPAVSESSLNENTSTQSDNDEGHGKHNQDTVHTVLSMGKQGSAFLPPKLDYNRSFACLPYTA
DAYYGGVLTGYPPHAVAHPQQNHTTNAPVMLPAEPAEEEPIYVNAKQYHAILRRRQTRAKLEAQNKLVKGRKPYLHESRHRHAMKRARGSGGRFLNTKQLQQTQQHQASGGSSSS
KVIGNSISSQSDPTPAPSTSTPSDTASASRANQDRTCFPVGLRPAMNFSAPGGEGAKLVR

>PvNF-YA12

MLLPSSSSSSASKGNSLRKTAIDNMRSTLSFDNKQPVFASQGIDYGQPIACISYPYNDSGSGGVWAAYGSRASAATVFHPQIAGGGTSARIPLPLELAENEPIYVNPKQYHGILRRRQFRA
KLEAQNKLVKARKPYLHESRHLHAMKRARGSGGRFLNTKQLQQQLRNASTRSTTNGTSSSGSTHLRLGGGTARDQTMLAPKAMPPQDNSKKAVSSAPGLTVTQMLRRDDTFFQHP
SHHLSFSGHFGQASAQAGMHNGTQHRVPVMR

>PvNF-YA13

MLPPHLIENGTIMIQFGHQMPDYDSPATQSTSESHQEVSGMSEGSLNDHNDQSGNHDGYSKSDENKMMSALSLGNPETAYAHPKPDRSQSFAISYPYADSYYGGAVAAYGQHAIMHP
QLVGMMPSSRVPLPIEPAAEEPIYVNAKQYHAILRRRQLRAKLEAENKLVKSRKPYLHESRHLHAMKRARGTSGRFLNTKQQSDSPGGSSDAQRMPANGGLFTKHEHSHPPGDRHY
HARGGA

>PvNF-YA14

MMSFKGHEGLGQVAAGSQASHVAAGAPLPWWAGPQLLYGESAPLSPEETRRDGQFQVVPGAQGGTPDPAPPAATKRGSPEVLKFSVFQGNLESGGKGEKNPEHSTTIALQSPFPEY
NGRFEIGLGQSMVPSNYSCDDQCYGVLTTYGMRSMSGGRMLLPLNAPADAPIYVNPKQYEGILRRRRARAKAERENRLAKGRKPYLHESRHLHAMRRARGSGGRFLNTKKEGGGA
NANGAGKTVSARLATSPSTEPPRVPELGKGNNGSNPRCHSRSSVSSLSGSEVTSIYDHHDDHASRHQYGGMEPHLRAPSFFTPLPAIMDGDHGGAATIPSFKWVASDGCCELLKA

>PvNF-YA15

MESRPDGTNLVEPRGPGAALPSGGAAMQQPWWTSSGAGLGAVAPAVVAPGSGAGISLSSSPVGSSGGAGAAKGAASDESSGDSRRSGEPKDGSAGQEKNHATSQMPALMSEYLTPY
SQLELNQSIGSAAYQYPDPYYAGMIPPYGTQALAHFQLPGLSHSRMPLPLEVSEEPVYVNAKQYHGILRRRQSRAKAELEKKVVKTRKPYLHESRHQHAMRRARGNGGRFLNTKKSD
NGTPNGNAEPKKGDQNSEHLRVPPDLLQLRQNEA

>PvNF-YA16

MMSFRSHEGFGQVPAGASSNGPAVPWWAPAPQLLLYGEALGQGKVPPEASPCREARFQVAPGAQAPLGPPVPPPPPAKAAAERGLPEVLKFSVAQGKGEKGSGHSATAALPSPFAIY
NGHFELGLGQSTVSANNLYVDQHCGLLSPYPMGATPGGCMRIPLNMPTEAPIYVNAKQYEGILRRRRARAKAERENRLVKARKPYLHESRHLHALRRARGSGGRFLNTKKESNSKD
AVGDGKAMFSNPLMRQVASPSSEIQQSDLGNPSSVSSLSGSEVSSIYDHEDVDHYHSFDHLRTPFFTPLPSIMDSEHGVGIPFKWPTASEGCCDLLRA

>PvNF-YA17

MTSVVHSVSGDHRAEDQHQQQKQAEPEDQQEAPVTSSDSQPTVGTPSADYVTPYAPHDMAHAMGQYAYPNIDPYYGSLYAAYGGQPMMHPPLVGMHPTGLPLPTDAIEEPVYVN
AKQYNAILRRRQSRAKAESERKLVKGRKPYLHESRHQHALKRARGAGGRFLNSKPDDKEENSDSSHKEKQNGVAPHKSGQPSTPPSPNGASSANQADSHQ

>PvNF-YB01

MPDSDKESGWPSNAEFSSPREQDRFLPIANVSRIMKMALPANAKISKDAKETVQECVSEFISFITGEASDKCQREKRKTINGDDLLWAMTTLGFEDYIEPLKLYLHKFRELEGEKVASGA
AGSSGSGSQPQRETTPSAHNGAGGAVGYGIYGAGAGAGGGSGMIMMMGQPMYNSPPGASGYPQPPHHQMVMAAKGGAYGHGGGSSPSPPGLGRQDRL

>PvNF-YB02

MKSRKGYGGQQGHLLSPVGSPPSDNESGAAAAAGCGSSAGYAGGGDSPAKELDRFLPIANVSRIMKRSLPANAKISKEAKETVQECVSEFISFVTGEASDKCQREKRKTINGDDLLWA
MTTLGFEAYVGPLKSYLNRYREAEGEKAAVGGARHGDGAADEGPLGAGAVAAAAPADRAGHHDAGGSMDVGLLMGVGAGGFGAGGGTSSYYSAAAGRAYGGDGSKVLEFDGE
EENGRGMQRGFGAGHLHGAVQL

>PvNF-YB03

MSTDPQGPKPRAPCAMPPEHAAVAKEAMVTEPLVPSTEPVMIREQDRLLPVANVSRIMRHALPPHAKISDDAKEMIQDCVSEFISFVTGEANERCHTEHRKTVTAEDLVWAMNRLGF
DDYVRPLNAFLQRMREIEGGGSGRGSRRGSSLVALQGAQTLRPAYPDAPQGYAVRPVPRPVPVPASSVASRFGSRHLQLPGGQRSMVPYYGGAAAFRAVGSHGGFYADEASSSDEAPP
AARRA

GSRRY

>PvNF-YB04

MADAPASPGGGGGSHESGSPRGGAGGGGGGVREQDRFLPIANISRIMKKAIPANGKIAKDAKETVQECVSEFISFITSEASDKCQREKRKTINGDDLLWAMATLGFEDYIEPLKVYLQK
YREMEGDSKLTAKTGDGSIKKDALGHGGASSSATQGMGQQGAYNQGMGYMQPQYHNGDISN

>PvNF-YB05

MSEAESAPETGGGSYGGKEQDRYLPIANIGRIMRRAVPENGKIAKDAKESVQECVSEFISFITSEASDKCMKEKRKTINGEDLIWSMGTLGFEEYVEPLKHYLNLYRETEGDTKGSKSSD
HTGKKEIVLNGEPGSSDSLHG

>PvNF-YB06

MPDSDNDSGGPSNAGGELSSPREQDRFLPIANVSRIMKKALPANAKISKDAKETVQECVSEFISFITGEASDKCQREKRKTINGDDLLWAMTTLGFEDYVEPLKHYLHKFREIEGERAA
ASSGASGSAAAQQQQGDVARGAANAAGYGAPGAGGMMMMMGQPMYGSPQQQPPPQQQQQQHQQHHMAMGGRGGYGHQGGGGGSSSSSGLGRQDRA

>PvNF-YB07

MPDSDNESGGPSNAEFSSPREQDRFLPIANVSRIMKKALPANAKISKDAKETVQECVSEFISFITGEASDKCQREKRKTINGDDLLWAMTTLGFEDYIEPLKLYLHKFRELEGEKVASGA
AGSSGSASQPQRETTPSAHNGAAGAVGYGMYGAGAGAGGGSGMIMMMGQPMYGSPPGASGYPQPPHHHMVMGAKGGAYGHGGGSSPSLSGLGRQDRL

>PvNF-YB08

MSENFNFTHFAQQRQPQNLSSASGSTTSKDDNSEAGHGNLLPIANVGRIMKEVLPPQAKISKRAKETIQECATEFVGFVTGEASERCRRERRKTINGDDICHAMRSLGLDHYADAMR
RYLQRYRESEELTAALNSGGIQIDVRAELSIFRGDDEQRDRN

>PvNF-YB09

MADHHGQGGRGGPGAEEIKEQDRLLPIANVGRIMKQILPPNAKISKEAKETMQECVSEFISFVTGEASDKCHKEKRKTVNGDDVCWAFGALGFDDYVDPMRRYLHKYRELEGDRA
AAAASSRGGGGSTSTSAGPDAGAGAGPSSGHFMFGAMDRSDNNSSSRPF

>PvNF-YB10

MADDGGSHEGGGGVREQDRFLPIANISRIMKKAVPANGKIAKDAKETLQECVSEFISFVTSEASDKCQKEKRKTINGDDLLWAMATLGFEEYVEPLKMYLHKYREMEGDSKLSTKAG
EGSVKKDAISPHGGTSSSSNQLVQHGVYNQGMGYMQPQYHNGDT

>PvNF-YB11

MDPASGFLPSGAANGTAAAGGSGQQHQAPPPIREQDRLMQIANVIRIMRRVLPSHAKISDDAKETIQECVSEYISFITGEANERCQREQRKTITAEDVLWAMSRLGFDDYVEPLSVYLH
RYREFEGEARGVVGLGVPGAARGGDHHHHPSMALKSRAPGAMSPPHHHDMQLHASMYGGPPAMPPPPGGPHHGHGFVGMPHHHGGGGGGGGGGHQSYPYEAAYAGEHAM
AAYYGSAAAYGAPGSGSSGGSASRTPQGGSFEHQHPFASYK

>PvNF-YB12

MDPASGFLPSGAANGTAAAGGGGQQQAPPPIREQDRLMPIANVIRIMRRVLPSHAKISDDAKETIQECVSEYISFITGEANERCQREQRKTITAEDVLWAMSRLGFDDYVEPLSVYLHR
YREFEGEARGVVGLGAPRGGDHHHHHSSMAPHHHDMQLHASMYGPPAVPPSGAPHHGHGLVSMPHHGGGGHQYLPYPYEAAYGGEHAMAAYYGSAAAYGAPGNGGSGDGSG
GSASRTRQGGSFEHPHPFASYK

>PvNF-YB13

MPDSDNDSGGPSNAGGELSSPREQDRFLPIANVSRIMKKALPANAKISKDAKETVQECVSEFISFITGEASDKCQREKRKTINGDDLLWAMTTLGFEDYVEPLKHYLHKFREIEGERAA
ASSGASGSAAAQQQGDVARGATNAGGYAGYSAGGMMMMGQPMYGSPPQQQQQQPPPPQQQHQQHHMAMGGRGGYGHQGGGGSSSSSGLGRQDRA

>PvNF-YB14

MSENFDFTGFAQQGQPQPQNLSRDDNSRAGHDNLLPIANVGRIMKDVLPPQAKISKHAKETIQECATEFIGFVTGEASERCRRERRKTINGDDICHAMRSLGLDHYANAMRRYLQRY
RESEELAAALNSGGGGIQIDVRAELSIFRGDEQQGKN

>PvNF-YB15

MADDGGSHEGGGGGGGVREQDRFLPIANISRIMKKAVPANGKIAKDAKETLQECVSEFISFVTSEASDKCQKEKRKTINGDDLLWAMATLGFEEYVEPLKMYLHKYREMEGDSKLST
KAGEGSVKKDAISPHGGTSSSSNQLVQHGVYNQGMGYMQPQYYNGDT

>PvNF-YB16

MADAPASPGGGGGSHESGSPKGGGGGGGGGVREQDRFLPIANISRIMKKAIPANGKIAKDAKETVQECVSEFISFITSEASDKCQREKRKTINGDDLLWAMATLGFEDYIEPLKVYLQK
YREMEGDSKLTAKAGDGSIKKDALGHGGASSSATQGMGQQGAYNQGMGYMQPQYHNGDISN

>PvNF-YB17

MSEAESAPETGGGSYGGKEQDRYLPIANIGRIMRRAVPDNGKIAKDAKESVQECVSEFISFITSEASDKCMKEKRKTINGDDLIWSLGTLGFEEYVEPLKHYLKLYREGDTKGSKSSDHT
GKKEIVLIGEPGSSLDGV

>PvNF-YB18

MSTDPQGPKPWAPCAMAPERAAVAKEAMSTEPLVPGTEPVIRGQDRLMPVANVSRIMRHALPPHAKISDGAKEMIQDCVSEFISFVTGEANERCHTEHRKTVTAEDIVWAMNRLGF
DDYIRPLNTFLQRMREIEGGDGAGGSGSGRGSRRGSSLVALHGAQTMRPAYPDAPQGYAVRPVIPRPVPVPASNAVASRFGSRYQLPGGQRSMAPYYGGAAFRAGGNRGGFYGDEAS
SSNEAPTARRARSRHY

>PvNF-YC01

MEPKSTTPPPAPVMGAPVAYPPPPAAAYAAGPYAHAPAAALYPPPPPPPPPHPAAAAVQQQLGPAAQQQLNLFWAEQFREVEATTDFKNHNLPLARIKKIMKADEDVRMIAAEAPV
VFARACEMFILELTHRGWAHAEENKRRTLQKSDIAAAVARTEVFDFLVDIVPRDEAKDAEAADAAGMGAGIPHPAAGMPAADPMGYYYVQPQ

>PvNF-YC02

MDQYSQEINNDDGATSGVTPGSYPLNTFLQPAALESVPLLSATAAFLPVPASQGAAAYPATILSSFSNQQSSQTNAFEAATNVAGGTPAAFALTPYNHQPEALASLPPSSSAGTTRLPSA
AGSSSSQVAGAGAAYQASIPVPLSSQHQAALFQKFQQLQQHQLQYQEQQLQALWTGQLVEAEQATDLKVHNLPLARIKKIMKADEDVKMIAGEVPVLFAKACEMFILELTLRSWLH
AEGAKRRTLQRSDVASAIIANEMFDFLMDVSPTEEQNGNGVAPPQQTATMPPATFPIPFPMYANQQPVTFMWPPTEYQQPQQRQQQNSDGGQAE

>PvNF-YC03

MFWAEQYREIEATTDFKNHNLPLARIKKIMKADEDVRMIAAEAPVVFARACEMFILELTHRGWAHAEENKRRTLQKSDIAAAIARTEVFDFLVDIVPRDDAKDADAAAAAAAAAA
AAGIPRPAAGVPATDPLAYYYVPQQ

>PvNF-YC04

MRQARPYSGMFCGGVSARTGPHALPLARIKKIMKRSAGETADGGARMISGEAPVVFSKACELFIAELTRRAWAATLEGKRRTVHKEDVATAVQKTDLFDFLVDVVMADAGGGGHA
AAGQYDDDDGALE

>PvNF-YC05

MDQYSRMINNDEGATSGVTPGSYPLTTYLQPAALESVPPSSAAGAAFLPVPPASQGAASYPPATMLSSFSNQQSSHTNAFQAATNVGTPAAFALTPYNHQPEALASVPPSSSSAGSSFFL
PPAAGSHGAGAAYQASIPVPLSSQNQAAFQRFQQLQQHQQQYQQQQLQALWTGQLVEAEQATDLKVHNLPLARIKKIMKADEDVKMIAAEVPVVFAKACEMFILELTLRSWLQAE
WSKRRTLQRSDVAAAIIANEMFDFLMDVSPTEEQNGGAPPPPQTATMPATFPMQHVPAFPMYANQQPAASFMWPPTEYQQQQQQQRQQQNSDGRQAE

>PvNF-YC06

MEPKSTTPLPAPVMGAPVAYPPPPAAAYAAGPYAHAPAAALYPPPPPPPPPHPSAAAVGQQLGPAAQQQLNLFWAEQFREVEATTDFKNHNLPLARIKKIMKADEDVRMIAAEAPV
VFARACEMFILELTHRGWAHAEENKRRTLQKSDIAAAVARTEVFDFLVDIVPRDEAKDAEAAAAAGMGAGIPHPAAGMPAADPMGYYYVQPQ

>PvNF-YC07

MEPSSQPQPVMGVATGGSQAYPAAASYAPPAMVPGAPAVVPPGSQPAAPFANPAQLSAQHQMVYQQAQQFHQQLQQQQQQQLREFWTTQMDEIEQTTDFKNHTLPLARIKKIMK
ADEDVRMISAEAPVVFAKACEVFILELTLRSWMHTEENKRRTLQKNDIAAAITRTDIYDFLVDIIPRDEMKEEGLGLPRVGLPPAVGAPGDSYPPYYYMTAQQMPPGGGMVYGGQQG
HPVTYMWQQPQGHEEEPPEEQQQQQRST

>PvNF-YC08

MDNQPLPYSTGQPPATGGAPVPGVPGAAGPPPVPHHHLLQQQQAQLQAFWAYQRQEAERASASDFKNHQLPLARIKKIMKADEDVRMISAEAPVLFAKACELFILELTIRSWLHAE
ENKRRTLQRNDVAAAIARTDVFDFLVDIVPREEAKEEPGSALGFAAAAGAGAVGSGAAGGAPAAGMPYYYPPMGQPAPMMPAWHVPAWDPAWQQGAAADVDQSGSFGEEGQG
FAAGHGGGAASFPPAAPPSSE

>PvNF-YC09

MEPSSQPQPVMGVATAGSQAYPAAAAYAPPAMVPGAPAVVPPGSQPAAPFPNPAQLSAQHQMVYQQAQQFHQQLQQQQQQQLREFWTTQMDEIEQTTDFKNHTLPLARIKKIMK
ADEDVRMISAEAPVVFAKACEVFILELTLRSWMHTEENKRRTLQKNDIAAAITRTDIYDFLVDIIPRDEMKEEGLGLQRVGLPPAVGAPTDSYPPYYYMTAQQMPPGGGMIYGDQQG
HPVTYMWQQPQGQDEEPPEEQRQQRSTLK

>PvNF-YC10

MDNQPLPYSTGQPPATGGAPVPGVPGAAGPPPVPHHHLLQQQQAQLQAFWAYQRQEAERASASDFKNHQLPLARIKKIMKADEDVRMISAEAPVLFAKACELFILELTIRSWLHAE
ENKRRTLQRNDVAAAIARTDVFDFLVDIVPREEAKEEPGSALGFAAAAGAGAVGGGAPGGAPAAGMPYYYPPMGQPAPMMPAWHVPAWDPAWQQGAAADVDQSGSFGEEGQG
FAGGHGGGAVSFPPPAPPSSE

>PvNF-YC11

MEPTSQPQPAIGVVAYPPAATIISPSAVAAAGLQPGQAFAANPAQMSAQHQVVYQQAQQFHLQLQQQQQRQLQQFWAERLADIEQTTDFKNHTLPLARIKKIMKADEDVRMISAEA
PVVFAKACEIFILELTLRSWMHTEENKRRTLQKNDIAAAITRTDIYDFLVDIVPRDEMKEEGVGLPRAGLPPMGAPADAHPYYYMPQQQVPGGGMVYGTQQGHPVTYLWQEPQEQQ
EPPPEEQQPLPGSG

>PvNF-YC12

MRQARPYSGIFCGGVSARTGPHALPLARIKKIMKRSAGETADGGARMISGEAPVVFSKACELFIAELTRRAWAATLEGKRRTVHKEDVATAVQKTDLFDFLVDVVMADAGGGGHAA
AGQYDDDDGALE

>AtNF-YA01

MDKKVSFTSSVAHSTPPYLSTSISWGLPTKSNGVTESLSLKVVDARPERLINTKNISFQDQDSSSTLSSAQSSNDVTSSGDDNPSRQISFLAHSDVCKGFEETQRKRFAIKSGSSTAGIADIH
SSPSKANFSFHYADPHFGGLMPAAYLPQATIWNPQMTRVPLPFDLIENEPVFVNAKQFHAIMRRRQQRAKLEAQNKLIKARKPYLHESRHVHALKRPRGSGGRFLNTKKLQESTDPK
QDMPIQQQHATGNMSRFVLYQLQNSNDCDCSTTSRSDITSASDSVNLFGHSEFLISDCPSQTNPTMYVHGQSNDMHGGRNTHHFSVHI

>AtNF-YA02

MTSSIHELSDNIGSHEKQEQRDSHFQPPIPSARNYESIVTSLVYSDPGTTNSMAPGQYPYPDPYYRSIFAPPPQPYTGLMGVQQQGVPLPSDAVEEPVFVNAKQYHGILRRRQSRARLES
QNKVIKSRKPYLHESRHLHAIRRPRGCGGRFLNAKKEDEHHEDSSHEEKSNLSAGKSAMAASSGTS

>AtNF-YA03

MQVFQRKEDSSWGNSMPTTNSNIQGSESFSLTKDMIMSTTQLPAMKHSGLQLQNQDSTSSQSTEEESGGGEVASFGEYKRYGCSIVNNNLSGYIENLGKPIENYTKSITTSSMVSQDSVF
PAPTSGQISWSLQCAETSHFNGFLAPEYASTPTALPHLEMMGLVSSRVPLPHHIQENEPIFVNAKQYHAILRRRKHRAKLEAQNKLIKCRKPYLHESRHLHALKRARGSGGRFLNTKK
LQESSNSLCSSQMANGQNFSMSPHGGGSGIGSSSISPSSNSNCINMFQNPQFRFSGYPSTHHASALMSGT

>AtNF-YA04

MMHQMLNKKDSATHSTLPYLNTSISWGVVPTDSVANRRGSAESLSLKVDSRPGHIQTTKQISFQDQDSSSTQSTGQSYTEVASSGDDNPSRQISFSAKSGSEITQRKGFASNPKQGSMTG
FPNIHFAPAQANFSFHYADPHYGGLLAATYLPQAPTCNPQMVSMIPGRVPLPAELTETDPVFVNAKQYHAIMRRRQQRAKLEAQNKLIRARKPYLHESRHVHALKRPRGSGGRFLN
TKKLLQESEQAAAREQEQDKLGQQVNRKTNMSRFEAHMLQNNKDRSSTTSGSDITSVSDGADIFGHTEFQFSGFPTPINRAMLVHGQSNDMHGGGDMHHFSVHI

>AtNF-YA05

MTSSVHELSDNNESHAKKERPDSQTRPQVPSGRSSESIDTNSVYSEPMAHGLYPYPDPYYRSVFAQQAYLPHPYPGVQLQLMGMQQPGVPLQCDAVEEPVFVNAKQYHGILRRRQSR
AKLEARNRAIKAKKPYMHESRHLHAIRRPRGCGGRFLNAKKENGDHKEEEEATSDENTSEASSSLRSEKLAMATSGPNGRS

>AtNF-YA06

MAMQTVREGLFSAPQTSWWTAFGSQPLAPESLAGDSDSFAGVKVGSVGETGQRVDKQSNSATHLAFSLGDVKSPRLVPKPHGATFSMQSPCLELGFSQPPIYTKYPYGEQQYYGVVS
AYGSQSRVMLPLNMETEDSTIYVNSKQYHGIIRRRQSRAKAAAVLDQKKLSSRCRKPYMHHSRHLHALRRPRGSGGRFLNTKSQNLENSGTNAKKGDGSMQIQSQPKPQQSNSQNS
EVVHPENGTMNLSNGLNVSGSEVTSMNYFLSSPVHSLGGMVMPSKWIAAAAAMDNGCCNFKT

>AtNF-YA07

MQEFHSSKDSLPCPATSWDNSVFTNSNVQGSSSLTDNNTLSLTMEMKQTGFQMQHYDSSSTQSTGGESYSEVASLSEPTNRYGHNIVVTHLSGYKENPENPIGSHSISKVSQDSVVLPIE AASWPLHGNVTPHFNGFLSFPYASQHTVQHPQIRGLVPSRMPLPHNIPENEPIFVNAKQYQAILRRRERRAKLEAQNKLIKVRKPYLHESRHLHALKRVRGSGGRFLNTKKHQESNSS LSPPFLIPPHVFKNSPGKFRQMDISRGGVVSSVSTTSCSDITGNNNDMFQQNPQFRFSGYPSNHHVSVLM

>AtNF-YA08

MGIEDMHSKSDSGGNKVDSEVHGTVSSSINSLNPWHRAAAACNANSSVEAGDKSSKSIALALESNGSKSPSNRDNTVNKESQVTTSPQSAGDYSDKNQESLHHGITQPPPHPQLVGH TVGWASSNPYQDPYYAGVMGAYGHHPLGFVPYGGMPHSRMPLPPEMAQEPVFVNAKQYQAILRRRQARAKAELEKKLIKSRKPYLHESRHQHAMRRPRGTGGRFAKKTNTEASK RKAEEKSNGHVTQSPSSSNSDQGEAWNGDYRTPQGDEMQSSAYKRREEGECSGQQWNSLSSNHPSQARL

AIK

>AtNF-YA09

MQTEELLSPPQTPWWNAFGSQPLTTESLSGEASDSFTGVKAVTTEAEQGVVDKQTSTTLFTFSPGGEKSSRDVPKPHVAFAMQSACFEFGFAQPMMYTKHPHVEQYYGVVSAYGSQRS SGRVMIPLKMETEEDGTIYVNSKQYHGIIRRRQSRAKAEKLSRCRKPYMHHSRHLHAMRRPRGSGGRFLNTKTADAAKQSKPSNSQSSEVFHPENETINSSREANESNLSDSAVTSMD YFLSSSAYSPGGMVMPIKWNAAAMDIGCCKLNI

>AtNF-YA10

MQSKPGRENEEEVNNHHAVQQPMMYAEPWWKNNSFGVVPQARPSGIPSNSSSLDCPNGSESNDVHSASEDGALNGENDGTWKDSQAATSSRSVDNHGMEGNDPALSIRNMHDQ PLVQPPELVGHYIACVPNPYQDPYYGGLMGAYGHQQLGFRPYLGMPRERTALPLDMAQEPVYVNAKQYEGILRRRKARAKAELERKVIRDRKPYLHESRHKHAMRRARASGGRFA KKSEVEAGEDAGGRDRERGSATNSSGSEQVETDSNETLNSSGAP

>AtNF-YB01

MTDEDRLLPIANVGRLMKQILPSNAKISKEAKQTVQECATEFISFVTCEASEKCHRENRKTVNGDDIWWALSTLGLDNYADAVGRHLHKYREAERERTEHNKGSNDSGNEKETNTRS DVQNQSTKFIRVVEKGSSSSAR

>AtNF-YB02

MERGAPFSHYQLPKSISELNLDQHSNNPTPMTSSVVVAGAGDKNNGIVVQQQPPCVAREQDQYMPIANVIRIMRKTLPSHAKISDDAKETIQECVSEYISFVTGEANERCQREQRKTIT AEDILWAMSKLGFDNYVDPLTVFINRYREIETDRGSALRGEPPSLRQTYGGNGIGFHGPSHGLPPPGPYGYGMLDQSMVMGGGRYYQNGSSGQDESSVGGGSSSSINGMPAFDHYGQ YK

>AtNF-YB03

MTEESPEEDHGSPGVAETNPGSPSSKTNNNNNNNNKEQDRFLPIANVGRIMKKVLPGNGKISKDAKETVQECVSEFISFVTGEASDKCQREKRKTINGDDIIWAITTLGFEDYVAPLKVY LCKYRDTEGEKVNSPKQQQQRQQQQQIQQQNHHNYQFQEQDQNNNNMSCTSYISHHHPSPFLPVDHQPFPNIAFSPKSLQKQFPQQHDNNIDSIHW

>AtNF-YB04

MAESQAKSPGGCGSHESGGDQSPRSLHVREQDRFLPIANISRIMKRGLPANGKIAKDAKEIVQECVSEFISFVTSEASDKCQREKRKTINGDDLLWAMATLGFEDYMEPLKVYLMRYRE MEGDTKGSAKGGDPNAKKDGQSSQNGQFSQLAHQGPYGNSQAQQHMMVPMPGTD

>AtNF-YB05

MAESQAKSPGGCGSHESGGDQSPRSLHVREQDRFLPIANISRIMKRGLPANGKIAKDAKEIVQECVSEFISFVTSEASDKCQREKRKTINGDDLLWAMATLGFEDYMEPLKVYLMRYRE MEGDTKGSAKGGDPNAKKDGQSSQNGQFSQLAHQGPYGNSQAQQHMMVPMPGTD

>AtNF-YB06

MAESQAKSPGGCGSHESGGDQSPRSLHVREQDRFLPIANISRIMKRGLPANGKIAKDAKEIVQECVSEFISFVTSEASDKCQREKRKTINGDDLLWAMATLGFEDYMEPLKVYLMRYRE MEGDTKGSAKGGDPNAKKDGQSSQNGQFSQLAHQGPYGNSQAQQHMMVPMPGTD

>AtNF-YB07

MADTPSSPAGDGGESGGSVREQDRYLPIANISRIMKKALPPNGKIGKDAKDTVQECVSEFISFITSEASDKCQKEKRKTVNGDDLLWAMATLGFEDYLEPLKIYLARYRELEGDNKGSG KSGDGSNRDAGGGVSGEEMPSW

>AtNF-YB08

MAGNYHSFQNPIPRYQNYNFGSSSSNHQHEHDGLVVVVEDQQQEESMMVKEQDRLLPIANVGRIMKNILPANAKVSKEAKETMQECVSEFISFVTGEASDKCHKEKRKTVNGDDIC WAMANLGFDDYAAQLKKYLHRYRVLEGEKPNHHGKGGPKSSPDN

>AtNF-YB09

MAESQTGGGGGGSHESGGDQSPRSLNVREQDRFLPIANISRIMKRGLPLNGKIAKDAKETMQECVSEFISFVTSEASDKCQREKRKTINGDDLLWAMATLGFEDYIDPLKVYLMRYRE MEGDTKGSGKGGESSAKRDGQPSQVSQFSQVPQQGSFSQGPYGNSQGSNMMVQMPGTE

>AtNF-YB10

MADSDNDSGGHKDGGNASTREQDRFLPIANVSRIMKKALPANAKISKDAKETVQECVSEFISFITGEASDKCQREKRKTINGDDLLWAMTTLGFEDYVEPLKVYLQKYREVEGEKTTT AGRQGDKEGGGGGGGAGSGSGGAPMYGGGMVTTMGHQFSHHFS

>AtNF-YB11

MDPMDIVGKSKEDASLPKATMTKIIKEMLPADVRVARDAQDLLIECCVEFINLISSESNEVCNKEDKRTIAPEHVLKALQVLGFGEYVEEVYAAYEQHKYETMQDSQRSVKMNSGAE MTEEEAAAEQQRMFAEARARMNGGVTVPQPEQLEEPQQQQQTSLQS

>AtNF-YB12

MDPMDIVGKSKEDASLPKATMTKIIKEMLPPDVRVARDAQDLLIECCVEFINLVSSESNDVCNKEDKRTIAPEHVLKALQVLGFGEYIEEVYAAYEQHKYETMQDTQRSVKWNPGAQ MTEEEAAAEQQRMFAEARARMNGGVSVPQPEHPETDQRSPQS

>AtNF-YB13

MGDSDRDSGGGQNGNNQNGQSSLSPREQDRFLPIANVSRIMKKALPANAKISKDAKETMQECVSEFISFVTGEASDKCQKEKRKTINGDDLLWAMTTLGFEDYVEPLKVYLQRFREIE GERTGLGRPQTGGEVGEHQRDAVGDGGGFYGGGGGMQYHQHHQFLHQQNHMYGATGGGSDSGGGAASGRTRT

>AtNF-YB14

MERGGFHGYRKLSVNNTTPSPPGLAANFLMAEGSMRPPEFNQPNKTSNGGEEECTVREQDRFMPIANVIRIMRRILPAHAKISDDSKETIQECVSEYISFITGEANERCQREQRKTITAE DVLWAMSKLGFDDYIEPLTLYLHRYRELEGERGVSCSAGSVSMTNGLVVKRPNGTMTEYGAYGPVPGIHMAQYHYRHQNGFVFSGNEPNSKMSGSSSGASGARVEVFPTQQHKY

>AtNF-YC01

MVSSKKPKEKKARSDVVVNKASGRSKRSSGSRTKKTSNKVNIVKKKPEIYEISESSSSDSVEEAIRGDEAKKSNGVVSKRGNGKSVGIPTKTSKNREEDDGGAEDAKIKFPMNRIRRIMR SDNSAPQIMQDAVFLVNKATEMFIERFSEEAYDSSVKDKKKFIHYKHLSSVVSNDQRYEFLADSVPEKLKAEAALEEWERGMTDAG

>AtNF-YC02

MDQQDHGQSGAMNYGTNPYQTNPMSTTAATVAGGAAQPGQLAFHQIHQQQQQQLAQQLQAFWENQFKEIEKTTDFKNHSLPLARIKKIMKADEDVRMISAEAPVVFARACE
MFILELTLRSWNHTEENKRRTLQKNDIAAAVTRTDIFDFLVDIVPREDLRDEVLGSIPRGTVPEAAAAGYPYGYLPAGTAPIGNPGMVMGNPGGAYPPNPYMGQPMWQQQAPDQPD
QEN

>AtNF-YC03

MDQQGQSSAMNYGSNPYQTNAMTTTPTGSDHPAYHQIHQQQQQQLTQQLQSFWETQFKEIEKTTDFKNHSLPLARIKKIMKADEDVRMISAEAPVVFARACEMFILELTLRSWNHT
EENKRRTLQKNDIAAAVTRTDIFDFLVDIVPREDLRDEVLGGVGAEAATAAGYPYGYLPPGTAPIGNPGMVMGNPGAYPPNPYMGQPMWQQPGPEQQDPDN

>AtNF-YC04

MEQSEEGQQQQQQGVMDYVPPHAYQSGPVNAASHMAFQQAHHFHHHHQQQQQQQLQMFWANQMQEIEHTTDFKNHTLPLARIKKIMKADEDVRMISAEAPVIFAKACEMFIL
ELTLRAWIHTEENKRRTLQKNDIAAAISRTDVFDFLVDIIPRDELKEEGLGVTKGTIPSVVGSPPYYYLQQQGMMQHWPQEQHPDES

>AtNF-YC05

MDTNNQQPPPSAAGIPPPPPGTTISAAGGGASYHHLLQQQQQQLQLFWTYQRQEIEQVNDFKNHQLPLARIKKIMKADEDVRMISAEAPILFAKACELFILELTIRSWLHAEENKRRT
LQKNDIAAAITRTDIFDFLVDIVPRDEIKDEAAVLGGGMVVAPTASGVPYYYPPMGQPAGPGGMMIGRPAMDPNGVYVQPPSQAWQSVWQTSTGTGDDVSYGSGGSSGQGNLDGQ
G

>AtNF-YC06

MENNNGNNQLPPKGNEQLKSFWSKEMEGNLDFKNHDLPITRIKKIMKYDPDVTMIASEAPILLSKACEMFIMDLTMRSWLHAQESKRVTLQKSNVDAAVAQTVIFDFLLDDDIEVK
RESVAAAADPVAMPPIDDGELPPGMVIGTPVCCSLGIHQPQPQMQAWPGAWTSVSGEEEEARGKKGGDDGN

>AtNF-YC07

MRRPKSSHVRMEPVAPRSHNTMPMLDQFRSNHPETSKIEGVSSLDTALKVFWNNQREQLGNFAGQTHLPLSRVRKILKSDPEVKKISCDVPALFSKACEYFILEVTLRAWMHTQSCTR
ETIRRCDIFQAVKNSGTYDFLIDRVPFGPHCVTHQGVQPPAEMILPDMNVPIDMDQIEEENMMEERSVGFDLNCDLQ

>AtNF-YC08

MEEEEGSIRPEFPIGRVKKIMKLDKDINKINSEALHVITYSTELFLHFLAEKSAVVTAEKKRKTVNLDHLRIAVKRHQPTSDFLLDSLPLPAQPVKHTKSVSDKKIPAPPIGTRRIDDFFSKG
KAKTDSA

>AtNF-YC09

MEENNGNNNHYLPQPSSSQLPPPPLYYQSMPLPSYSLPLPYSPQMRNYWIAQMGNATDVKHHAFPLTRIKKIMKSNPEVNMVTAEAPVLISKACEMLILDLTMRSWLHTVEGGRQTL
KRSDTLTRSDISAATTRSFKFTFLGDVVPRDPSVVTDDPVLHPDGEVLPPGTVIGYPVFDCNGVYASPPQMQEWPAVPGDGEEAAGEIGGSSGGN

>AtNF-YC10

MAENNNNNGDNMNNDNHQQPPSYSQLPPMASSNPQLRNYWIEQMETVSDFKNRQLPLARIKKIMKADPDVHMVSAEAPIIFAKACEMFIVDLTMRSWLKAEENKRHTLQKSDIS
NAVASSFTYDFLLDVVPKDESIATADPGFVAMPHPDGGGVPQYYYPPGVVMGTPMVGSGMYAPSQAWPAAAGDGEDDAEDNGGNGGGN

>AtNF-YC11

MENNNNNNHQQPPKDNEQLKSFWSKGMEGDLNVKNHEFPISRIKRIMKFDPDVSMIAAEAPNLLSKACEMFVMDLTMRSWLHAQESNRLTIRKSDVDAVVSQTVIFDFLRDDVPKD
EGEPVVAAADPVDDVADHVAVPDLNNEELPPGTVIGTPVCYGLGIHAPHPQMPGAWTEEDATGANGGNGGN

>AtNF-YC12

MDNNNNNNNNQQPPPTSVYPPGSAVTTVIPPPPSGSASIVTGGGATYHHLLQQQQQQLQMFWTYQRQEIEQVNDFKNHQLPLARIKKIMKADEDVRMISAEAPILFAKACELFILELTI
RSWLHAEENKRRTLQKNDIAAAITRTDIFDFLVDIVPREEIKEEEDAASALGGGGMVAPAASGVPYYYPPMGQPAVPGGMMIGRPAMDPSGVYAQPPSQAWQSVWQNSAGGGDDV
SYGSGGSSGHGNLDSQG
